# Epitranscriptomic silencing of the ZC3H13/m^6^A axis orchestrates immunosuppressive microenvironment remodeling in renal cell carcinoma via CSF2-mediated MDSCs recruitment

**DOI:** 10.1186/s13046-026-03742-2

**Published:** 2026-05-20

**Authors:** Jinxiu Zheng, Xueting Zhao, Qing Men, Mengyi Zhou, Yuxin Che, Shenglu Liu, Likun Zan, Lingzhi Guo, Ying Shao, Shuhua Gao, Yanjie Ma, Xiaofeng Liu, Lijun Yang, Tao Yang

**Affiliations:** 1 School of Basic Medicine, Institute of Cancer Biology, Shanxi Medical University, Jinzhong, China; 2 Department of Biochemistry & Molecular Biology, Shanxi Medical University, Jinzhong, China; 3 School of Medical Sciences, Shanxi Medical University, Jinzhong, China; 4 Shanxi Center of Technology Innovation for Biomolecular Imaging & Precision Oncology, Taiyuan, China; 5 Sichuan Institute of Industrial Technology, Deyang, China; 6 Department of Pathology, The Affiliated Cancer Hospital of Shanxi Medical University, Taiyuan, China; 7 Laboratory of Morphology, Shanxi Medical University, Jinzhong, China; 8 Department of Pathology, Shanxi University of Medicine, Fenyang, China; 9 Department of Urology, First Hospital of Shanxi Medical University, Taiyuan, China; 10 Key Laboratory of Carcinogenesis and Translational Research (Ministry of Education/Beijing), Peking University Cancer Hospital, Beijing, China; 11 Department of Pharmacology, Shanxi University of Medicine, Fenyang, China; 12 Ministry of Education Key laboratory of Cellular Physiology, Shanxi Medical University, Jinzhong, China; 13 Key laboratory of Digestive Disease & Organ Transplantation in Shanxi Province, The First Hospital of Shanxi Medical University, Taiyuan, Shanxi China; 14 Ministry of Education Key Laboratory of Coal Environmental Pathogenicity and Prevention, School of Public Health, Shanxi Medical University, Jinzhong, China; 15 Collaborative Innovation Center for Molecular Imaging of Precision Medicine, Shanxi Medical University, Taiyuan, China

**Keywords:** Renal cell carcinoma, ZC3H13, CSF2, UNC5CL, MDSCs

## Abstract

**Background:**

The immunosuppressive tumor microenvironment (TME) is a cardinal driver of immune escape and resistance to immunotherapy in renal cell carcinoma (RCC). The N6-methyladenosine (m^6^A), the most prevalent RNA modification, critically governs tumor aggressiveness and TME reprogramming. We aimed to exploit whether and how tumor-intrinsic m^6^A modification driven by ZC3H13 (zinc finger CCCH-type containing 13) can dictate the immune landscape of RCC.

**Methods:**

Loss- and gain-of-function studies were performed in vitro and in allograft tumor model. Tumor-infiltrating immune cells were profiled with flow cytometry and immunostaining. The pivotal cytokine mediated by ZC3H13 depletion was identified through an integrated analysis of RNA-seq and PCR array. The molecular target of ZC3H13 was elucidated through integrated m^6^A sequencing and RNA sequencing.

**Results:**

Low ZC3H13 expression was an independent predictor of poor prognosis in RCC. Functionally, ZC3H13 silencing enhanced the malignant behaviors of RCC cells and drove an immunosuppressive microenvironment characterized by aberrant recruitment of myeloid-derived suppressor cells (MDSCs). Mechanistically, ZC3H13 promoted m^6^A methylation on UNC5CL (UNC-5 Family C-Terminal Like) mRNA, facilitating YTHDC1-mediated stabilization of UNC5CL transcripts, which subsequently suppressed the NF-κB-CSF2 signaling axis, ultimately inhibiting MDSCs accumulation. Furthermore, therapeutic targeting CSF2 in combination with anti-PD1 exerts stronger antitumor effects in RCC by synergistically reversing the MDSCs mediated immunosuppressive microenvironment.

**Conclusion:**

We identified that ZC3H13 loss unleashes the NF-κB-CSF2 signaling cascade via m^6^A-dependent silencing of UNC5CL, converting the RCC milieu into an MDSCs-dominated immunosuppressive niche. Targeting CSF2 combined with anti-PD1 may represent a novel therapeutic strategy to achieve better efficacy in RCC.

**Supplementary Information:**

The online version contains supplementary material available at 10.1186/s13046-026-03742-2.

## Background

Renal cell carcinoma (RCC) represents one of the most formidable challenges in urologic oncology, characterized by its aggressive nature and a significant capacity for immune evasion [1]. The latest statistics from the National Cancer Center indicate a daunting trend, with approximately 73,700 new RCC cases diagnosed in China in 2022, accompanied by an estimated 24,000 deaths attributable to this malignancy [2]. Alarmingly, the incidence of RCC continues its upward trajectory, rising from 0.36 to 0.62 [3], while the five-year survival rate for patients diagnosed with advanced stages of the disease remains dishearteningly low, dipping below 20% [4].

The advent of immune checkpoint blockade (ICB), a novel therapeutic approach, has transformed the treatment landscape for numerous cancers. Although advanced RCC is often heavily infiltrated by CD8⁺ T cells and is considered an immunologically “hot” tumor, its response to immunotherapy remains suboptimal—a phenomenon described as the “hot tumor, cold effect” paradox [1, 5]. This paradox is compounded by the observation that high levels of CD8⁺ T cell infiltration are paradoxically associated with a poorer prognosis in this disease [6]. The core mechanism underlying this discrepancy lies in the complex immunosuppressive network within the tumor microenvironment (TME) [1]. This network comprises abundant immunosuppressive cells, such as regulatory T cells (Treg), M2-type tumor-associated macrophages, and myeloid-derived suppressor cells (MDSCs), alongside inhibitory signaling molecules. Together, they establish an “immunological fortress” that effectively dampens cytotoxic T cell function [7]. Emerging evidence has illuminated the pivotal role of the TME in shaping RCC progression and therapeutic resistance [8, 9]. The dynamics within the TME significantly influence tumor immune escape, thereby impeding the efficacy of immunotherapy strategies and directly correlating with impacting patient survival outcomes [10, 11]. Thus, a key and urgent scientific challenge is to identify strategies that can reverse this immunosuppressive TME in RCC.

Cancer cell-intrinsic features are pivotal in driving both tumor biology and the composition of the immune landscape, ultimately influencing the efficacy of immunotherapeutic approaches such as ICB [12, 13]. Among the numerous pathways implicated in these processes, N6-methyladenosine (m^6^A) modification, a widespread and critical post-transcriptional modification of mRNA [14, 15], stands out as a key regulator. ZC3H13 (zinc finger CCCH-type containing 13), a crucial component of the m^6^A methyltransferase complex, interacts with target RNA sequences through its CCCH zinc finger domains, orchestrating gene transcription and translation [16, 17]. The involvement of ZC3H13 in tumorigenesis has been substantiated, linking its activity to various malignancies [17, 18]. Notably, a growing body of literature highlights correlations between ZC3H13 expression and immune cell infiltration levels within the TME [19, 20]. Furthermore, emerging data have suggested that m^6^A modification may modulate the responsiveness to ICB, illustrating its relevance in therapeutic contexts [21]. However, the precise role of ZC3H13 in RCC pathogenesis and the regulatory mechanisms influencing the tumor immune landscape remain elusive.

In this study, we unveil the critical association between tumor-intrinsic ZC3H13 expression and RCC progression, along with its impact on MDSCs accumulation within the RCC microenvironment. Through our investigations, we delineate a signaling cascade whereby epitranscriptomic silencing of the ZC3H13/m^6^A axis leads to the suppression of UNC5CL expression, which subsequently activates an NF-κB-CSF2 signaling pathway that recruits MDSCs, resulting in T-cell dysfunction and tumor progression. Importantly, we demonstrate that combining CSF2 targeting with anti-PD1 blockade yields superior therapeutic efficacy, positioning this pathway as a potential therapeutic target in RCC. Collectively, our findings provide valuable insights into the intricate interplay between epitranscriptomics and the immune microenvironment, paving the way for novel therapeutic strategies aimed at improving outcomes for RCC patients.

## Methods

### Cell culture

Human embryonic kidney cell line HEK293T and RCC cell lines (786-O, ACHN, A-498, OS-RC-2) were obtained from the National Biomedical Cell-Line Resource in 2021. Murine RCC cell line Renca was purchased from the Procell system in 2022. 786-O, OS-RC-2 and Renca were cultured in RPMI 1640 growth medium (BOSTER); ACHN, A-498 and HEK293T cells were maintained in Minimum Essential Medium (MEM, Solarbio), all culture medium were supplemented with 10% fetal bovine serum (FBS, Cellmax) and 1% penicillin/streptomycin (Solarbio). All cells were incubated at a 37 °C in a humidified incubator (Thermo) with 5% CO_2_.

### Bioinformatics analysis

The transcriptomic data and corresponding clinical information for kidney renal clear cell carcinoma (KIRC) were obtained from The Cancer Genome Atlas (TCGA) database, a publicly accessible resource. Samples lacking gene expression or survival data were excluded from the analysis. Differential expression of m^6^A regulatory genes between tumor and normal samples was assessed using Partial Least Squares Discriminant Analysis (PLS-DA) on the OmicStudio platform (https://www.omicstudio.cn/tool). The list of m^6^A regulatory genes was shown in Table S1.

### m^6^A colorimetric quantification

The global m^6^A level in total RNA was quantified using the EpiQuik™ m^6^A RNA Methylation Quantification Kit (Colorimetric) (EpiGentek) in strict accordance with the manufacturer’s protocol. Briefly, 200 ng RNA was added to each well, followed by the sequential addition of the capture antibody and detection antibody. The m^6^A content was quantified colorimetrically by measuring the absorbance at 450 nm, with concentrations calculated from a standard curve.

### Dot blot

Total RNA was extracted from cell pellets using the M5 Universal RNA Mini Kit (Mei5bio). RNA samples were adjusted to concentrations of 80 ng/mL, 40 ng/mL, and 20 ng/mL, denatured at 95 °C for 5 min in a metal bath, and 10 µL of each sample was spotted onto a nylon membrane (Beyotime). Membranes were cross-linked under 254 nm UV light for 1 h, blocked with 5% skim milk for 1 h at room temperature, and incubated with m^6^A antibodies overnight at 4 °C. After washing, membranes were incubated with HRP-conjugated secondary antibodies for 1 h at room temperature and developed using an Ultra-Sensitive ECL Chemiluminescence Kit (SEVEN).

### RNA interference

Small interfering RNAs (siRNAs) targeting human YTHDF1, YTHDF2, YTHDF3, YTHDC1, YTHDC2, IGF2BP1, IGF2BP2, and IGF2BP3, along with negative control siRNA (siCtrl), were synthesized by Haixing Biosciences. Transient transfection was performed using Lipofectamine 3000 reagent (Thermo) or NanoTrans Transfection Reagent 3000 (CYTOCH) according to the manufacturer’s protocol. At 72 h post-transfection, cells were harvested for qPCR or western blot analysis to assess knockdown efficiency. All siRNA sequences are listed in Table S2.

### Construction of stable knockdown and overexpressed cells

Lentiviruses expressing human/mouse sh*ZC3H13* or shCtrl were purchased from Tsingke Biotech. Lentiviruses for mouse *ZC3H13* overexpression or empty vector control, overexpression plasmids (pLV-puro-flag-UNC5CL) and vector plasmid were purchased from Shanghai Genechem Co.,Ltd. All shRNA target sequences are listed in Table S3.

### Quantitative real-time PCR (qPCR)

Total RNA was isolated from cell pellets using the M5 Universal RNA Mini Kit (Mei5bio) following the manufacturer’s protocol. Complementary DNA (cDNA) was synthesized from RNA using HiFiScript gDNA Removal RT MasterMix (CWBIO). Quantitative real-time PCR was performed with TB Green^®^ Premix Ex Taq (TaKaRa) using gene-specific primers. Relative mRNA expression levels were calculated via the 2^−∆∆Ct^ method. All qPCR primers are listed in Table S4.

### Western blot

Total protein was extracted from cultured cells using RIPA lysis buffer (Beyotime) supplemented with protease inhibitor cocktail (PIC), phenylmethanesulfonyl fluoride (PMSF), and phosphatase inhibitors (BOSTER). Protein concentration was quantified with a BCA Protein Assay Kit (NCM). Samples were denatured at 100℃ for 10 min, and 40–50 µg of protein per well was loaded onto 7.5%–10% SDS-PAGE gels for electrophoresis. Separated proteins were transferred to nitrocellulose (NC) membranes (MERCK), blocked with 5% skim milk for 1 h at room temperature, and incubated with primary antibodies overnight at 4°C. After washing, membranes were incubated with horseradish peroxidase (HRP)-conjugated secondary antibodies for 1 h at room temperature. Protein bands were visualized using an Ultra-Sensitive ECL Chemiluminescence Kit (SEVEN). The antibody list is shown in Table S5.

### Clinical samples and immunohistochemical (IHC)

A cohort of 60 RCC tissue samples from Shanxi Provincial Cancer Hospital was constructed into tissue microarrays with patient consent. IHC staining was performed following standard procedures: sections were deparaffinized, rehydrated, subjected to antigen retrieval, and blocked for endogenous peroxidase and nonspecific binding. They were then incubated with primary antibodies at 4°C overnight, followed by HRP-conjugated secondary antibodies and DAB development, with final counterstaining and mounting. IHC score was calculated by multiplying the staining intensity (0–3) by the percentage of positive cells (0–4). All slides were independently evaluated by two pathologists. Antibody details are listed in Table S5.

### Cell viability and colony formation assay

Cell viability was determined using the Cell Counting Kit-8 (CCK-8) assay (BOSTER). Stable cells were seeded in 96-well plates at a density of 1,000 cells per well in 100 µL of culture medium. Absorbance was detected at 450 nm daily for 4 consecutive days. About colony formation assay, cells in the logarithmic growth phase were trypsinized and seeded into 6-well plates at a density of 2,000 cells per well. The cells were then cultured in complete medium for approximately 2 weeks. The colonies were washed twice with PBS, fixed with 4% paraformaldehyde, and stained with 0.1% crystal violet (Solarbio). The number of colonies was subsequently counted and photographed.

### Animals

All animal experiments were approved by the Institutional Animal Care and Use Committee of Shanxi Medical University. Four-week-old Balb/c female mice and NSG mice were purchased from SPF (Beijing) Biotechnology Co. Ltd. The mice were housed under specific pathogen-free (SPF) conditions with a 12-hour light/dark cycle, maintained at 18–22°C, and with 50–60% humidity. Cervical dislocation was used as the euthanasia method for all experimental mice.

### Subcutaneous syngeneic tumor models

Renca murine RCC cells stably expressing shCtrl and sh*Zc3h13* were subcutaneously injected into Balb/c and/or NSG mice (5 × 10^6^ cells per mouse). Tumor size was measured every three days, and the volume was calculated using the formula: Volume (mm^3^) = (longest diameter) × (shortest diameter)^2^ × 0.5. After three weeks or when the tumor burden reached the predefined endpoint, the mice were euthanized, and the tumors were harvested and weighed. Immune cell infiltration in the tumor microenvironment was subsequently analyzed by flow cytometry.

### ssGSEA and TIMER database analysis

For the bulk RNA‑seq data from the TCGA‑KIRC cohort, patients were stratified into high‑ and low‑ZC3H13 expression groups using the median expression level as the cutoff. Single‑sample gene set enrichment analysis (ssGSEA) was performed using the GSVA R package to estimate the relative infiltration levels of 23 immune cell types in each sample, with reference gene sets from MSigDB. The resulting ssGSEA score matrix was used for subsequent correlation analysis to characterize the immune functional status of the tumor microenvironment. Furthermore, the online TIMER database (https://cistrome.shinyapps.io/timer/), a well-validated web-based analytical platform, was employed to further validate and evaluate the potential correlation between ZC3H13 transcriptional expression level and the infiltration degree of MDSCs.

### Single‑cell RNA‑sequencing (scRNA‑seq) analysis

The single-cell RNA-sequencing dataset GSE159115 was downloaded from the Gene Expression Omnibus (GEO) database. After quality control, standard preprocessing and dimensionality reduction, reliable cell clustering and accurate cell type annotation were performed based on canonical cell lineage marker genes. Tumor cell clusters were first extracted using canonical epithelial markers (EPCAM, CA9), and the median cutoff for grouping was determined solely using ZC3H13 expression in these tumor cells. Subsequently, differential infiltration characteristics and compositional differences of immune cell subsets were quantitatively compared between the two subgroups.

### Multicolor flow cytometry

Tumor‑infiltrating immune cells from subcutaneous xenografts were profiled using multicolor flow cytometry. Tumor samples were excised into single-cell suspensions using Tumor Dissociation Kit (mouse) (Miltenyi Biotec) according to the manufacturer’s instructions. Cells were first stained with a fixable viability dye and then incubated with an Fc receptor blocking reagent for 15 min at room temperature. Subsequently, cells were stained with fluorochrome‑conjugated monoclonal antibodies against surface markers. After fixation and permeabilization, intracellular or intranuclear staining was performed using corresponding antibodies. Samples were acquired on a BD FACSCelesta flow cytometer (BD Biosciences), and data were analyzed with FlowJo software. Antibodies used are listed in Table S5.

### MDSCs isolation and MDSCs migration assay

Tumor-bearing mice were euthanized by cervical dislocation after orbital bleeding, followed by immersion in 75% ethanol for 3 min. Spleens were isolated under sterile conditions in a laminar flow hood, fully ground, and filtered to prepare single-cell suspensions. MDSCs were negatively isolated using the EasySep™ Mouse MDSC (CD11b^+^Gr1^+^) Isolation Kit (STEMCELL) and cultured in RPMI-1640 basic medium. For the migration assay, MDSCs (5 × 10^5^ cells/200 µL) were seeded into Transwell chambers, and shCtrl or sh*Zc3h13* Renca cells suspensions (1 × 10^6^ cells/600 µL) were added to the lower chamber. After 24 h of incubation, the number of migrated MDSCs in the lower chamber was counted.

### MDSCs differentiation assay

Bone marrow-derived cells (BMDCs) were isolated from the femurs of Balb/c mice. To assess tumor-induced MDSCs differentiation, naive BMDCs were co-cultured with conditioned medium from shCtrl or sh*Zc3h13* Renca cells. RPMI-1640 medium alone and medium supplemented with 40 ng/mL IL-6 and CSF2 (Peprotech) served as negative and positive controls, respectively. After 5 days of co-culture, cells were then harvested and analyzed by flow cytometry after staining with antibodies against CD45, CD11b, and Gr-1.

### MDSCs expansion assay

For the mouse MDSCs expansion assay, isolated MDSCs were labeled with CFSE (BD Biosciences). The labeled MDSCs were then co-cultured for 3 days with conditioned medium derived from shCtrl or sh*Zc3h13* Renca cells. Positive control wells were supplemented with 40 ng/mL IL-6 and CSF2, while negative controls contained RPMI-1640 medium alone. Proliferation was assessed by flow cytometry based on CFSE dilution.

### Antibody neutralization and immune checkpoint blockade

IgG isotype control (BE0089, Bio-X-Cell), anti-Gr-1 (BE0075, Bio-X-Cell), anti-mouse GM-CSF (BE0259, Bio-X-Cell), and anti-mouse PD1 antibody (BE0146, Bio-X-Cell) were purchased by Bio X Cell. When tumor size reached approximately 100 mm^3^, mice were treated with IgG control, anti-GM-CSF (250 µg/mouse every 3 days), anti-PD1 (200 µg/mouse every 3 days) or anti-Gr-1(150 µg/mouse every 3 days) via intraperitoneal injections.

### Enzyme linked immunosorbent assay (ELISA)

The concentrations of human and mouse CSF2 in conditioned media or serum were measured using Human and Mouse CSF2 ELISA Kits (MEIMIAN) according to the manufacturer’s instructions. Briefly, cell conditioned media with FBS was collected and centrifuged to remove cells or debris. Processed samples and standards were added to the antibody-precoated wells and incubated. After extensive washing, a biotinylated detection antibody was applied, followed by streptavidin-conjugated horseradish peroxidase (HRP). The signal was developed with TMB substrate, and the absorbance was measured at 450 nm.

### RNA sequencing (RNA-seq) and m^6^A-modified RNA immunoprecipitation sequencing (MeRIP-seq)

Total RNA was first extracted from ACHN cells with stable *ZC3H13* knockdown and corresponding vector-transfected cells. The quality and quantity of the RNA were assessed by Eppendorf BioPhotometer^®^ D30. Denaturing agarose gel electrophoresis was used to assess RNA integrity. Subsequently, MeRIP-seq and RNA-seq libraries were constructed and sequenced by RiboBio (Guangzhou, China). Differentially expressed genes and differentially methylated transcripts were identified using the thresholds of |log2(fold change)| > 1.5 and *P*-value < 0.05.

### MeRIP qPCR

The m^6^A-modified RNA fragments were immunoprecipitated using the BersinBio™ MeRIP Kit according to the manufacturer’s instructions. Briefly, total RNA was extracted from cells, and RNA integrity was confirmed by denaturing agarose gel electrophoresis. Qualified RNA was then fragmented to approximately 100 nucleotides. An anti-m^6^A antibody was conjugated to a mixture of protein A/G magnetic beads by overnight incubation at 4°C. The antibody-bead complex was then incubated with the fragmented RNA to enrich m^6^A-modified fragments. The enrichment of m^6^A on the UNC5CL/FOXJ1/UPK3B/TNXB transcript was quantified by qPCR using specific primers (Table S2). Relative enrichment of m^6^A was normalized to input: %Input = 2 ^(Ct^_IP​_^−(Ct^_input​_^−3))^.

### RNA immunoprecipitation (RIP)

The RIP assay was performed using a BersinBioTM RNA Immunoprecipitation (RIP) Kit according to the manufacturer’s instructions. Briefly, the corresponding cell lysates were incubated with beads coated with 5 µg of control IgG antibody, anti-ZC3H13 antibody or anti-YTHDC1 with rotation at 4°C overnight. Next, total RNA was extracted for the detection of UNC5CL expression by qPCR.

### RNA stability assay

Cells were seeded in 6-well plates at a uniform density. When cell confluence reached 80%, actinomycin D (Aladdin) was added to complete medium at a final concentration of 10 µg/mL. Cells were harvested at 0, 2, 4, 6 h after actinomycin D treatment. mRNA expression levels were determined by qPCR as described above.

### Nuclear and cytoplasmic protein extraction

The nuclear and cytoplasmic fractions were isolated using a commercially available kit (Nuclear and Cytoplasmic Protein Extraction Kit, Beyotime Biotechnology) according to the manufacturer’s instructions. In brief, cells were lysed in a cytoplasmic extraction buffer, and the supernatant (cytoplasmic fraction) was collected after centrifugation. The remaining nuclear pellet was then lysed in a nuclear extraction buffer to obtain the nuclear fraction. GAPDH or LaminB1 were served as loading controls for cytosolic or nuclear fraction, respectively.

### Cell immunofluorescence

Cells (786-O-shCtrl and 786-O-sh*ZC3H13*) were seeded on sterile coverslips in 6-well plates at 5 × 10^4^ cells per well and cultured for 24 h. After fixing with 4% paraformaldehyde for 30 min and permeabilizing with 0.2% Triton X-100 for 15 min at room temperature, cells were blocked with 5% BSA for 1 h, then incubated overnight at 4℃ with anti-p65 primary antibody (1:1500). After washing, cells were incubated with a fluorescent secondary antibody for 1 h in the dark, followed by DAPI staining for 5 min. Coverslips were mounted with anti-fade mounting medium, and images were acquired using a confocal microscope, with fluorescence intensity quantified by ImageJ software.

### Statistical analysis

Data are expressed as mean ± standard deviation (SD). Differences between two groups were analyzed using the unpaired Student’s t-test. Comparisons of growth curves were performed by repeated-measures analysis of variance (ANOVA). Survival analysis was conducted using the Kaplan–Meier method, and differences between curves were assessed with the log-rank test. Univariate and multivariate Cox proportional hazards regression models were employed to evaluate the prognostic significance of ZC3H13 expression. All statistical analyses were performed using GraphPad Prism (version 9.5) and SPSS (version 20.0). *P*-value < 0.05 was considered statistically significant.

## Results

### Reduced ZC3H13 expression correlates with unfavorable prognosis of patients with RCC

**Fig. 1:**
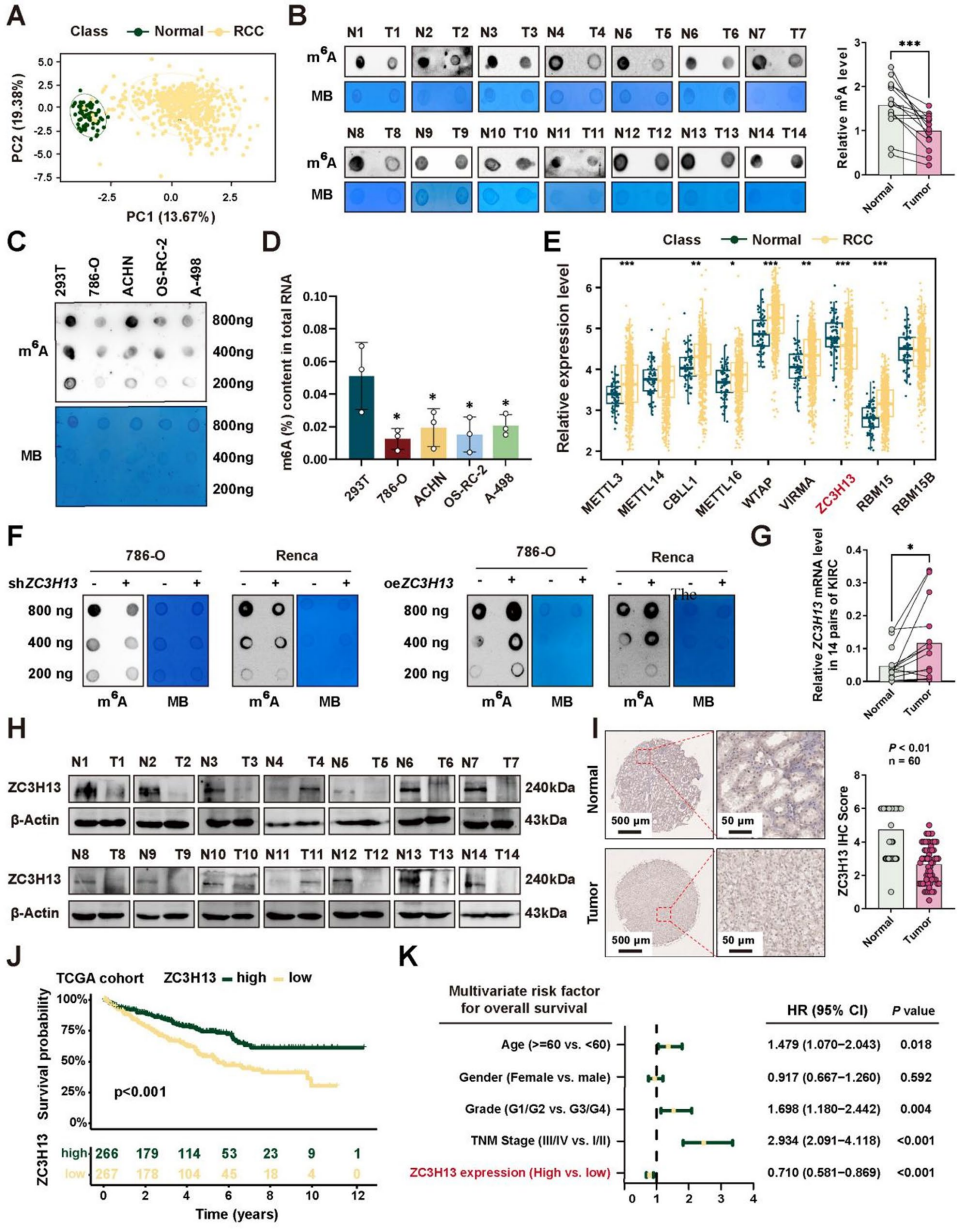
Low ZC3H13 expression correlates with poor prognosis of patients with RCC. **A** Partial Least Squares Discriminant Analysis (PLS-DA) of 30 m^6^A regulators in RCC tissues (*n* = 542) and normal tissues (*n* = 72). The list of m^6^A regulatory genes was shown in Table S1. **B** Global m^6^A levels in RCC and paired normal adjacent tissues was assessed by dot blot, with relative m^6^A contents on mRNA quantified (right panel). **C-D** Global m^6^A levels in 293T and human RCC cell lines (786-O, ACHN, OS-RC-2, A-498) was assessed by dot blot (C) and colorimetric ELISA-like assay via the m^6^A RNA methylation quantification kit (D). **E** Expression profiles of nine m^6^A methyltransferases (“writers”) in RCC and normal samples from the TCGA-KIRC cohort. **F** Effects of *ZC3H13* knockdown and overexpression on m^6^A modification levels, as determined by dot blot analysis. **G-H** The levels of ZC3H13 expression in RCC and paired normal adjacent tissues were detected by qPCR and western blot (*n* = 14); **I** Representative images and immunostaining scores of ZC3H13 IHC on formalin-fixed, paraffin-embedded sections of 60 RCC tissues and matched adjacent normal tissues. **J** Kaplan-Meier OS analysis of ZC3H13 expression in patients with RCC (log-rank test). **K** Multivariable Cox regression analysis of overall survival in the TCGA-KIRC cohort. Forest plot showing the hazard ratios (HR) and 95% confidence intervals (CI) for each clinical variable, including age (≥ 60 vs. <60), gender (female vs. male), grade (G1/G2 vs. G3/G4), TNM stage (III/IV vs. I/II), and ZC3H13 expression (high vs. low, stratified by the median expression level of ZC3H13). All histogram chart data are presented as the mean ± SD. Statistical analyses were performed by Graphpad Prism 9.5. ns, not significant, *P < 0.05, **P < 0.01

To elucidate the role of m^6^A modification in RCC, we first analyzed the expression profiles of 30 m^6^A-related regulatory factors in the TCGA-KIRC transcriptome data. PLS-DA revealed pronounced heterogeneity in the expression of these m^6^A regulatory factors between RCC samples and normal samples (Fig. 1A). Experimentally, both colorimetric ELISA via the m^6^A RNA methylation quantification kit and dot blot assays demonstrated significantly reduced m^6^A levels in RCC tissues (Fig. 1B) as well as RCC cell lines (Fig. 1C-D). Collectively, these data underscore that a potential alteration in RNA m^6^A profile during RCC development.

We therefore hypothesized that the global loss of m^6^A modification in RCC is driven by dysregulation expression of m^6^A regulatory genes. A systematic expression screen of known m^6^A “writer” genes (METTL3, METTL14, CBLL1, METTL16, WTAP, VIRMA, ZC3H13, RBM15, RBM15B) revealed ZC3H13 as the most strikingly downregulated factor across TCGA cohorts (Fig. 1E), a finding which aligns with the previously observed global reduction in m^6^A modification levels (Fig. 1B-D). To address the role of ZC3H13 in m^6^A regulation, we examined global m^6^A levels in RCC cells via colorimetric ELISA and dot blot after manipulation of ZC3H13 expression (Supplementary Fig. 1, 2). The results revealed that global m^6^A level were decreased in *ZC3H13*-knockdown 786-O/ACHN/Renca cells, whereas overexpression of *ZC3H13* increased the total m^6^A level (Fig. 1F, Supplementary Fig. 3A-D).

Analysis of the 14 paired samples revealed a marked reduction in ZC3H13 mRNA and protein expression in primary RCC versus matched adjacent normal tissues (Fig. 1G-H). This finding was corroborated by IHC, which showed consistently low expression of ZC3H13 in RCC tissues (Fig. 1I). We further extended our findings to RCC cell lines (Supplementary Fig. 4A-B), indicating that ZC3H13 expression is downregulated in RCC cells. Moreover, a clear decreasing trend in ZC3H13 expression was observed with increasing tumor grade and stage of TCGA cohort (Supplementary Fig. 4C-D).

To further elucidate the clinical relevance of ZC3H13 in RCC, patients were stratified into high‑ZC3H13 and low‑ZC3H13 expression groups using the median ZC3H13 expression level in the TCGA‑KIRC dataset as the cutoff. Strikingly, survival analysis revealed that low ZC3H13 expression was associated with significantly shortened overall survival in RCC patients (Fig. 1J). Simultaneously, uni-variate Cox regression analysis revealed that Age, Grade, TNM stage and ZC3H13 expression were substantially associated with 5-year survival in patients with RCC (Supplementary Fig. 4E). Moreover, multivariate Cox regression analysis established ZC3H13 as an independent prognostic factor (Fig. 1K). Taken together, these results therefore identify ZC3H13 as a key prognostic factor in RCC, prompting us to investigate its functional significance.

### Dysregulation of ZC3H13 drives the immunosuppressive microenvironment remodeling by MDSCs

**Fig. 2:**
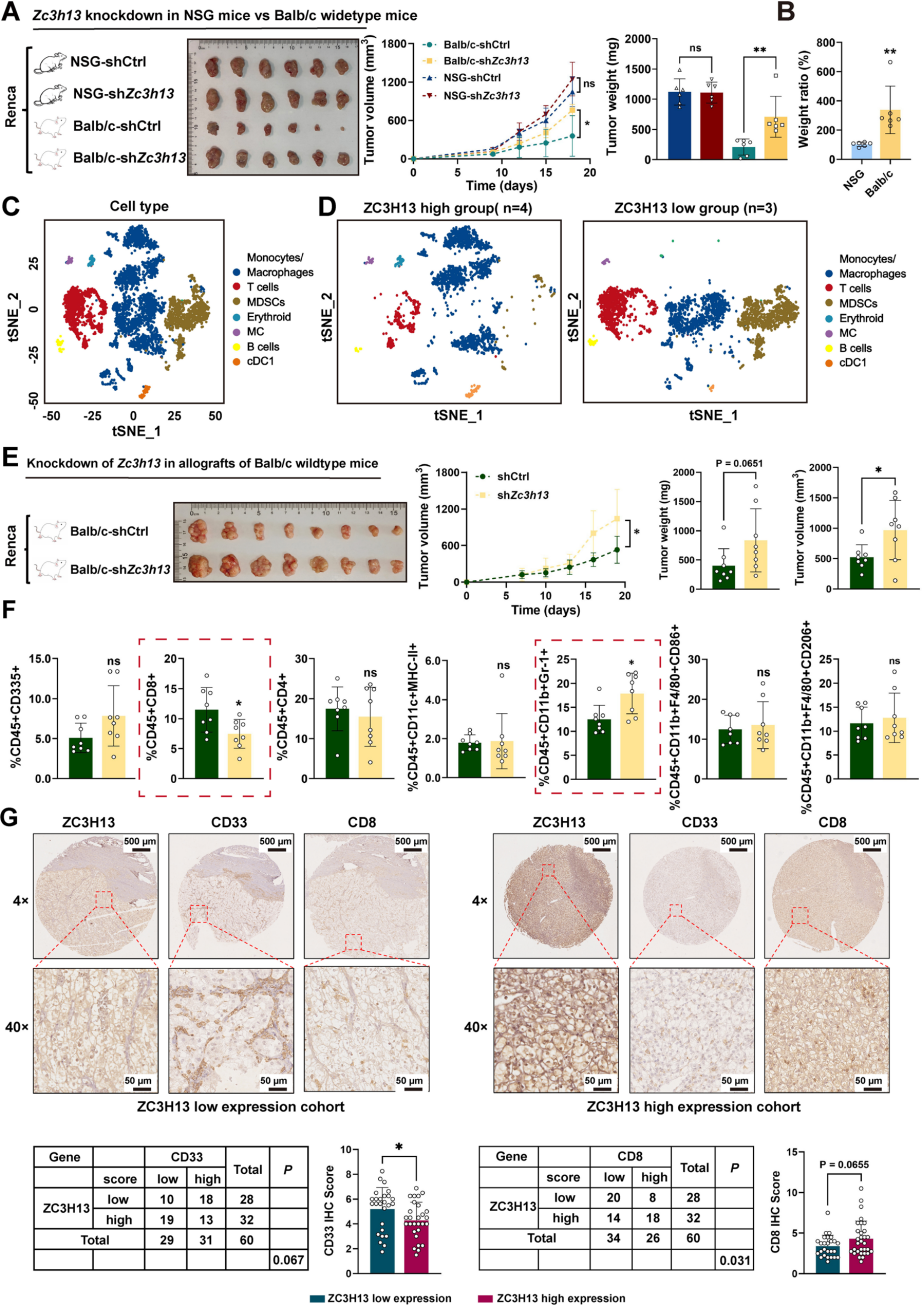
Dysregulation of ZC3H13 drives the immunosuppressive microenvironment remodeling by MDSCs. **A** Subcutaneous Renca tumor models in NSG and Balb/c mice: macroscopic tumor morphology images, tumor growth curves throughout the experimental period, and statistics of terminal tumor weights in the four mouse groups (*n* = 6); **B** Weight ratio between the two mouse groups, calculated as Weight ratio = sh*ZC3H13* (g) / shCtrl (g) ⋅ 100%. **C-D** The t-distributed stochastic neighbor embedding (t-SNE) plot isolated from RCC tissues from the GSE159115, with each cell color coded for cell type (B), and sample group (C). **E** Representative images of harvested tumor in each group, tumor volume and weight of Renca tumors with *Zc3h13* knockdown (*n* = 8). **F** Flow cytometry analysis of CD8^+^ T cell, NK cell, CD4^+^ T cell, dendritic cell, MDSCs, Tregs, M1 macrophage, M2 macrophage in Renca allografts (*n* = 8). **G** Representative IHC images of ZC3H13, CD8, and CD33 in human renal carcinoma tissues, correlation between ZC3H13 expression and CD8^+^ T cell and MDSCs infiltration percentage (*n* = 60). All histogram chart data are presented as the mean ± SD. Statistical analyses were performed by Graphpad Prism 9.5. ns, not significant, *P < 0.05, **P < 0.01

To investigate the function role of ZC3H13 in RCC, we performed gain- and loss-of-function studies in 786-O and ACHN human RCC cells. *ZC3H13* knockdown exerted a slight promotive effect on cell proliferation in vitro (Supplementary Fig. 5A, C), whereas, *ZC3H13* overexpression exerted the opposite effects (Supplementary Fig. 5B, D). To further assess ZC3H13 function in vivo, we established xenograft models by implanting Renca cells stably expressing control shRNA or sh*Zc3h13* into immunocompetent Balb/c mice or immunodeficient NSG mice. Interestingly, *Zc3h13* knockdown promoted significantly more aggressive tumor growth in Balb/c mice than in NSG mice (Fig. 2A-B, Supplementary Fig. 6A-B), suggesting that the tumor-suppressive role of ZC3H13 is mediated by a functional immune system. Taken together, these findings indicate that ZC3H13 expression may modulate antitumor immune responses in RCC.

To elucidate whether ZC3H13 exerts its tumor‑suppressive effects by remodeling the tumor immune microenvironment, we first analyzed a publicly available single-cell RNA-sequencing dataset of RCC (GSE159115). Samples were stratified into high-ZC3H13 and low-ZC3H13 expression groups based on the ZC3H13 level within malignant cells. Notably, tumor tissues with low ZC3H13 expression displayed a significantly higher infiltration of myeloid‑derived suppressor cells (MDSCs) relative to those with high ZC3H13 expression (Fig. 2C‑D; Supplementary Fig. 7A‑D). This finding preliminarily confirms an inverse correlation between ZC3H13 expression and MDSCs infiltration, suggesting that ZC3H13 may contribute to shaping an immunosuppressive tumor microenvironment by modulating MDSCs accumulation in RCC.

To functionally validate the above bioinformatic correlation and further gain insights into the cellular heterogeneity within the tumor microenvironment, flow cytometry was performed on tumor tissues from the sh*Zc3h13* and shCtrl groups. In an Renca syngeneic mice model, both tumor volume and weight were markedly increased by *Zc3h13* knockdown (Fig. 2E). Notably, we observed a marked accumulation of MDSCs (Fig. 2G), accompanied by a reduction of CD8^+^ T cells (Fig. 2F-G, Supplementary Fig. 8) in *Zc3h13*-knockdown tumors. To validate the clinical relevance of these observations, we performed IHC on human RCC samples to examine the correlation between ZC3H13 expression and the infiltration of MDSCs and CD8⁺ T cells. The results revealed that ZC3H13‑low expression tissues exhibited a significant increase in MDSCs abundance and a concurrent decrease in CD8⁺ T cell infiltration, which aligns closely with the findings from the animal models (Figs. 2H). Specifically, ssGSEA analysis also revealed that compared to the high ZC3H13 expression group, the low ZC3H13 expression group exhibited increased infiltration of MDSCs (Supplementary Fig. 9A), which was further validated using the TIMER database (Supplementary Fig. 9B). Collectively, these results strengthen the evidence that ZC3H13 expression modulates antitumor immune responses in RCC, potentially through regulating MDSCs within the tumor microenvironment.

**Fig. 3:**
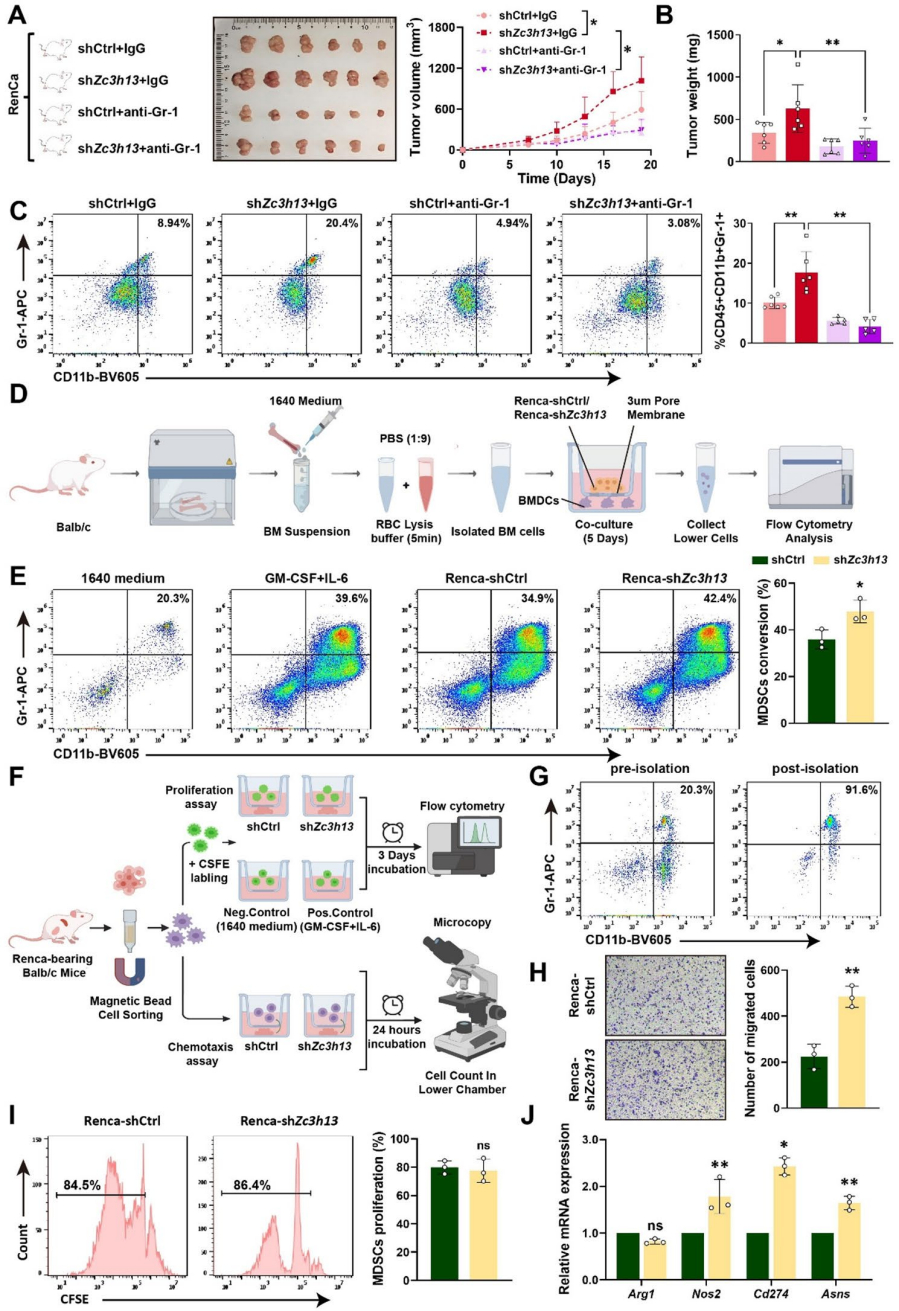
Low expressing of Zc3h13 induces the conversion, chemotaxis, and function of MDSCs. **A-B** Representative images of harvested tumor in each group, tumor volume and weight in Renca-shCtrl and Renca-sh*Zc3h13* syngeneic transplanted tumors treated with anti-Gr1 antibody or IgG control (*n* = 6); **(C)** Infiltration of MDSCs from the treatment groups described in (A) (*n* = 6 in shCtrl + IgG and sh*Zc3h13* + IgG group, *n* = 5 in shCtrl + anti-Gr-1 and sh*Zc3h13* + anti-Gr-1). **D** Schematic of BMDCs isolation from tumor-bearing mice and differentiation into MDSCs. **E** Representative flow cytometry plots showing the conversion of BMDCs into MDSCs under different culture conditions. **F** MDSCs were isolated from the spleens of tumor-bearing mice and used for CFSE proliferation and chemotaxis assays. **G** Flow cytometry analysis of the positive expression rate of MDSCs cells before and after sorting with magnetic beads. **H** Representative images and quantification analysis of MDSCs migration toward Renca shCtrl and sh*Zc3h13* cells. **I** Flow cytometry was performed after 3 d of coculture by gating on live cells to determine the percentage of MDSCs that diluted CFSE. **J** qPCR analysis of functional markers in MDSCs cultured with conditioned medium from Renca shCtrl or sh*Zc3h13* cells. All histogram chart data are presented as the mean ± SD. Statistical analyses were performed by Graphpad Prism 9.5. ns, not significant, *P < 0.05, **P < 0.01

We therefore hypothesized that knockdown of *Zc3h13* drives the establishment of an immunosuppressive tumor microenvironment characterized by MDSCs expansion. We sought to test this hypothesis by depleting MDSCs by anti–Gr-1 antibody in Renca allografts. We found that anti–Gr-1 treatment abolished the tumor-promoting effect of sh*Zc3h13* (Figs. 3A-B, supplementary Fig. 10A-C) by depleting MDSCs (Figs. 3C). Hence, *Zc3h13* regulates immune suppression via modulating MDSCs infiltration. The accumulation of MDSCs within the TME is orchestrated through three principal processes: conversion from BMDCs, recruitment by tumor-derived chemokines, and local proliferation. We further sought to identify the exact molecular mechanism underlying sh*ZC3H13*-driven MDSCs accumulation, namely whether ZC3H13 affects one or more of these processes (i.e., MDSCs recruitment, differentiation, and proliferation). To assess MDSCs differentiation, BMDCs from Balb/c mice were isolated and cocultured with conditioned medium from shCtrl and sh*Zc3h13* Renca cells (Fig. 3D). Results showed that sh*Zc3h13* cells induced a larger proportion of MDSCs than shCtrl cells (Fig. 3E). Then, to investigate whether ZC3H13 expression affects MDSCs chemotaxis, MDSCs were isolated from the spleens of tumor-bearing mice via magnetic bead-based sorting, with a purity of > 90% confirmed by flow cytometry (Fig. 3F-G). As shown in Fig. 3H, MDSCs migration assays revealed that *ZC3H13* knockdown significantly promoted MDSCs chemotaxis, as evidenced by increased MDSCs migration in the Renca-sh*Zc3h13* group versus control. For MDSCs expansion, CFSE-labeled purified murine MDSCs were cocultured with conditioned medium from shCtrl and sh*Zc3h13* Renca cells using transwell chambers. CFSE assay showed that sh*Zc3h13* had no effect on MDSCs expansion (Fig. 3I). We next asked if sh*Zc3h13* enhances MDSCs function. MDSCs were cultured in conditioned medium from shCtrl and sh*Zc3h13* Renca cells, revealing that markers of MDSCs function, including Nos2, CD274, Asns were all upregulated in the sh*Zc3h13* group (Fig. 3J). In summary, our findings demonstrated that *ZC3H13* loss orchestrates a pro-tumor immune microenvironment by promoting MDSCs conversion and chemotaxis, enhancing their immunosuppressive function.

### Knockdown of *ZC3H13* drives MDSCs accumulation by secreting CSF2

**Fig. 4:**
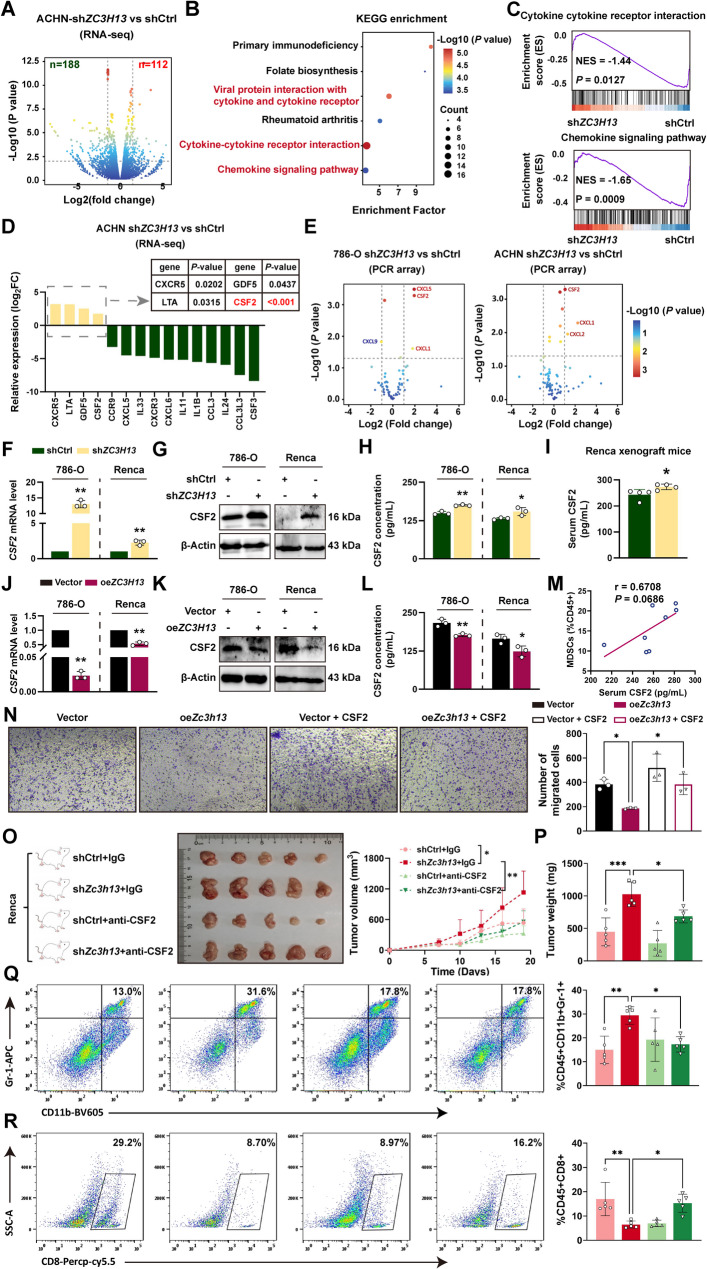
ZC3H13 regulates the secretion of CSF2 to modulate the tumor immune microenvironment. **A** Volcano plot of differentially expressed genes between 786-O-shCtrl and 786-O-shZC3H13 cells from RNA-seq. **B** KEGG pathway enrichment analysis of the differentially expressed genes. **C** GSEA enrichment plots for cytokine and cytokine receptor pathways and chemokine signaling pathways. **D** Changes in cytokine and chemokine expression levels between 786-O-shCtrl and 786-O-shZC3H13 cells from RNA-seq. **E** Volcano plot of differentially expressed cytokine/chemokine mRNA expression levels by PCR arrays in 786-O/ACHN cells. **F-G** The mRNA and protein expression levels of CSF2 following ZC3H13 knockdown in 786-O/Renca cells were detected by qPCR and western blot. **H** ELISA analysis of CSF2 secretion in the supernatant of 786-O/Renca cells with or without ZC3H13 knockdown. **I** Serum CSF2 protein concentration of Renca syngeneic mice models. **J-K** The mRNA and protein expression levels of CSF2 following ZC3H13 overexpression in 786-O/Renca cells. **L** ELISA analysis of CSF2 secretion in the supernatant of 786-O/Renca cells with or without ZC3H13 overexpression. **M** Correlation analysis of serum CSF2 and MDSCs levels in Renca syngeneic mice model. **N **Representative images and quantification analysis of MDSCs migration toward Renca vector and oeZc3h13 cells with or without recombinant CSF2 protein. **O-P** Representative photographs of resected tumors, quantitative measurements of tumor volume, and tumor weight of Renca-shCtrl and Renca-shZc3h13 allografts treated with anti-CSF2 or IgG. **Q-R** Flow cytometry analysis of tumor-infiltrating MDSCs and CD8+ T cells across the different treatment groups. All histogram chart data are presented as the mean ± SD. Statistical analyses were performed by Graphpad Prism 9.5. ns, not significant, *P < 0.05, **P < 0.01.

The generation and egress of MDSCs are promoted by tumor secreted cytokines and chemokines. To elucidate the factor(s) that mediate MDSCs accumulation, we performed RNA sequencing in *ZC3H13* knockdown (sh*ZC3H13*) and control (shCtrl) human RCC 786-O cells. In *ZC3H13* knockdown cells, 112 genes were upregulated and 188 genes were downregulated (|log2(fold change)| > 1.5, *P* < 0.05 ) (Fig. 4A). The top downregulated pathways were enriched in viral protein interaction with cytokine and cytokine receptor pathways and chemokine signaling pathways (Fig. 4B), which were confirmed by gene set enrichment analysis based on the KEGG database (Fig. 4C).

Among genes of cytokine-cytokine receptor interaction pathway, significant increase of C-X-C Motif Chemokine Receptor 5 (CXCR5), lymphotoxin alpha (LTA), growth differentiation factor 5 (GDF5) and colony stimulating factor 2 (CSF2) expression was found in ACHN-sh*ZC3H13* cells (Fig. 4D). To gain deeper insights into the expression profiles of cytokines and chemokines, we performed transcriptomic analysis using a 90-gene Human Cytokines & Chemokines PCR Array in 786-O and ACHN cells. In line with the RNA-seq findings, sh*ZC3H13* cells displayed elevated CSF2 expression relative to shCtrl cells (Fig. 4E). Furthermore, qPCR and western blot also confirmed that *ZC3H13* knockdown significantly increased CSF2 expression in 786-O/ACHN/Renca cells (Fig. 4F-G, Supplementary Fig. 11A-B). Moreover, ELISA assays revealed that *ZC3H13* knockdown increased CSF2 secretion in cell culture supernatants of 786-O/ACHN/Renca cells (Fig. 4H, Supplementary Fig. 12C), as well as in the serum of mice harboring Renca allografts (Fig. 4I). Conversely, ectopic expression of *ZC3H13* in 786-O/Renca exerted the opposite effect (Fig. 4J-L). These findings suggest that knockdown of *ZC3H13* promotes CSF2 expression and secretion in RCC.

CSF2 is a cytokine reported to promote RCC and is a regulator of MDSCs. Notably, a marked correlation between serum CSF2 and MDSCs levels was assesed in Renca syngeneic mice model (Fig. 4M). We thus hypothesized that sh*ZC3H13*-mediated CSF2 secretion might contribute to MDSCs recruitment and function. We next investigate the role of the ZC3H13-CSF2 axis in MDSCs chemotaxis using in vitro MDSCs migration assay. Overexpression of *Zc3h13* in Renca cells markedly impaired the chemotaxis of MDSCs toward the tumor cell-conditioned medium. Notably, addition of recombinant CSF2 protein rescued MSDCs migration in *Zc3h13* overexpression cells (Fig. 4N). We then extended our analysis to an in vivo model to validate that *ZC3H13* knockdown remodels the MDSCs-dominated immunosuppressive tumor microenvironment by upregulating the expression and secretion of CSF2, thereby facilitating the malignant progression of RCC. As shown in Fig. 4O-R, anti-CSF2 treatment effectively abrogated sh*ZC3H13*-induced MDSCs recruitment and RCC allografts growth in vivo, concomitant with restored CD8^+^ T infiltration in tumors. Collectively, our data indicated that sh*Zc3h13*-mediated CSF2 secretion might contribute to MDSCs recruitment and function, thereby promoting RCC progression.

### ZC3H13 increased UNC5CL mRNA levels through an m^6^A-YTHDC1 dependent mechanism

**Fig. 5:**
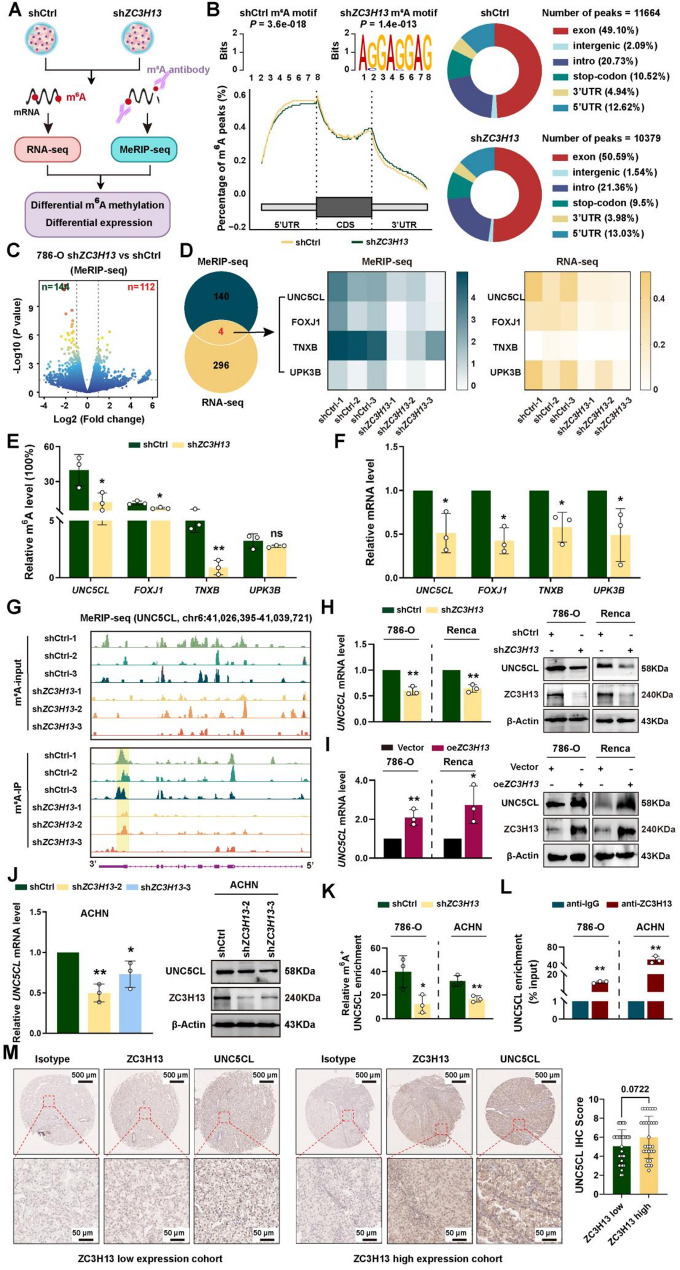
Identification of UNC5CL as a functional target of ZC3H13 in RCC. **A** Workflow of screening candidate target of ZC3H13. **B** Genomic distribution of m^6^A peaks and enriched m^6^A modification motifs identified by MEME analysis in ACHN-shCtrl and ACHN-sh*ZC3H13* cells. **C** Volcano plot of differential m^6^A methylated genes derived from MeRIP-seq. **D** Venn diagram showing the overlapped candidate genes between MeRIP-seq and RNA-seq. Heatmap of the overlapped candidate genes in MeRIP-seq and RNA-seq is shown. **E** MeRIP-qPCR analysis of m^6^A levels of four candidate gens in 786-O cells with or without *ZC3H13* knockdown. **F** qPCR analysis of mRNA levels of four candidate gens in 786-O cells with or without *ZC3H13* knockdown. **G** IGV visualization of MeRIP-seq reads along *UNC5CL* mRNA. **H** mRNA and protein levels of UNC5CL following *ZC3H13* knockdown in 786-O/Renca cells. **I** mRNA and protein levels of UNC5CL following *ZC3H13* overexpression in 786-O/Renca cells. **J** mRNA and protein levels of UNC5CL following *ZC3H13* knockdown in ACHN cells. **K** MeRIP-qPCR analysis of m^6^A enrichment on *UNC5CL* mRNA in 786-O/ACHN cells upon *ZC3H13* knockdown. **L** RIP-qPCR analysis of ZC3H13 protein binding to *UNC5CL* mRNA in 786-O/ACHN cells. **M** Representative immunohistochemical (IHC) images of ZC3H13 and UNC5CL in human RCC tissue microarrays (left), comparison of UNC5CL IHC scores between the ZC3H13-low and ZC3H13-high groups (right). All histogram chart data are presented as the mean ± SD. Statistical analyses were performed by Graphpad Prism 9.5. ns, not significant, *P < 0.05, **P < 0.01

To decipher the mechanistic link between ZC3H13-dependent m^6^A modification and CSF2 expression, m^6^A MeRIP-seq and RNA-seq was conducted in ACHN-shCtrl and ACHN-sh*ZC3H13* cells (Fig. 5A). m^6^A MeRIP-seq profiling revealed that *ZC3H13* knockdown decreased the number of detectable m^6^A peaks compare with control, with 11,664 and 10,379 peaks identified in ACHN-shCtrl and ACHN-sh*ZC3H13* cells, displaying a predominant distribution in the vicinity of start and stop codons. The m^6^A consensus motif GGAC was identified (Fig. 5B). As such, 144 m^6^A peaks were significantly downregulated (|log(fold change)| > 1.5, *P* < 0.05) upon *ZC3H13* knockdown (Fig. 5C). Notably, no m^6^A peak was observed in CSF2, implying that CSF2 is not a direct target of ZC3H13.

To identify downstream targets of ZC3H13-m^6^A axis, we overlapped 300 differentially expressed transcription factors (RNA-seq) and 144 down-regulated m^6^A targets (m^6^A MeRIP-seq), which yielded four candidate ZC3H13 downstream targets (UNC5CL, FOXJ1, TNXB, UPK3B) (Fig. 5D). IGV visualization analysis showed that the m^6^A modification levels of the above four candidate genes were significantly reduced upon *ZC3H13* knockdown (Fig. 5G, Supplementary Fig. 12A). MeRIP-qPCR analysis confirmed that knockdown of *ZC3H13* in 786-O cells led to significantly decreased m^6^A levels of UNC5CL, FOXJ1, and TNXB (Fig. 5E). Moreover, qPCR analysis showed that *ZC3H13* knockdown significantly reduced transcript levels of UNC5CL, FOXJ1, TNXB, and UPK3B (Fig. 5F).

Notably, knockdown of *ZC3H13* inhibited UNC5CL (UNC-5 Family C-Terminal Like, also known as ZUD) expression in both 786-O/ACHN/Renca cells (Fig. 5H and J), whereas FOXJ1, TNXB and UPK3B protein levels showed no significant alterations (Supplementary Fig. 12B). Thus, UNC5CL may be a direct target of ZC3H13 in RCC cells. Consistently, ectopic expression of *ZC3H13* in 786-O/Renca cells increased UNC5CL mRNA and protein expression (Fig. 5I). Furthermore, MeRIP-qPCR demonstrated that knockdown of *ZC3H13* decreased the m^6^A level of *UNC5CL* mRNA in 786-O/ACHN cells (Fig. 5K). We next asked whether ZC3H13 could directly bind to *UNC5CL* mRNA. To this end, RIP with anti-ZC3H13 antibody was performed in 786-O/ACHN cells, revealing a direct interaction between ZC3H13 and *UNC5CL* mRNA (Fig. 5L). Finally, IHC revealed a significant positive correlation between ZC3H13 and UNC5CL expression in RCC tissues (Fig. 5M). All these results collectively suggest that UNC5CL is a critical downstream target of ZC3H13 in RCC.

**Fig. 6:**
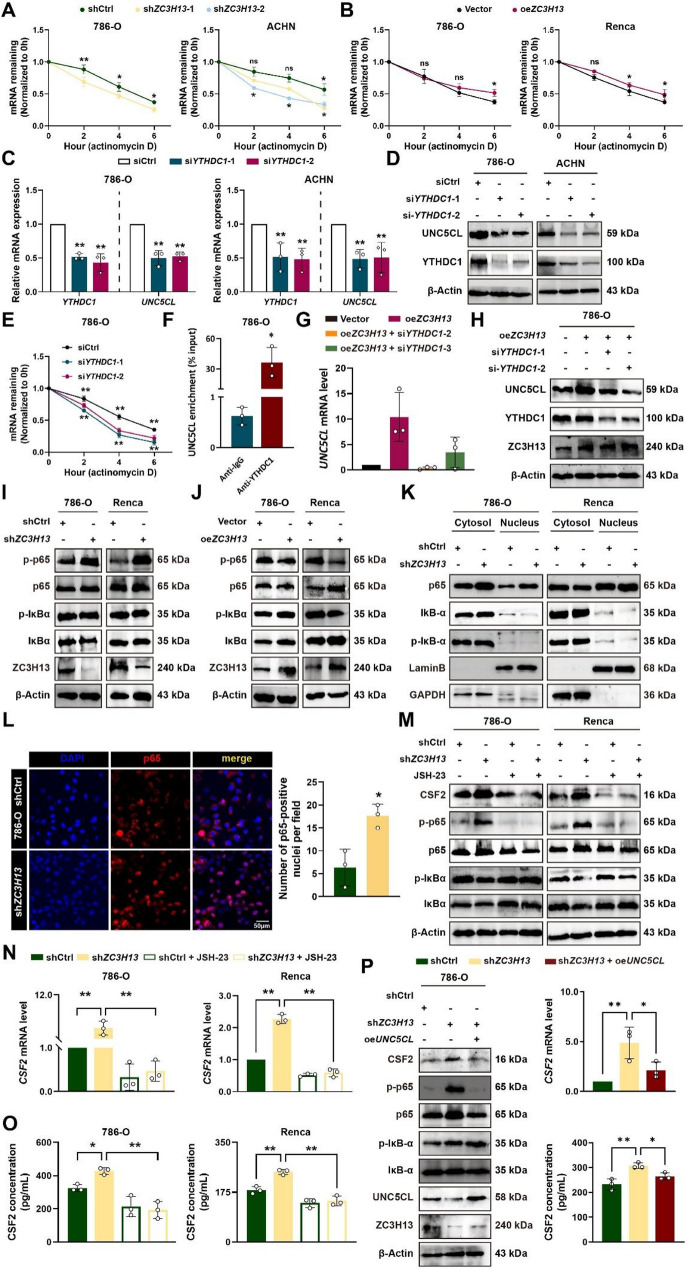
Loss of *ZC3H13* activates the NF-κB-CSF2 signaling axis via UNC5CL. **A-B** RNA stability assay for UNC5CL mRNA in cells with *ZC3H13* knockdown or overexpression after treatment with actinomycin D. **C-D** UNC5CL mRNA and protein levels upon *YTHDC1* knockdown in 786-O and ACHN cells by qPCR and western blot. **E** RNA stability assay for UNC5CL mRNA in cells with *YTHDC1* knockdown after treatment with actinomycin D. **F** RIP-qPCR analysis of YTHDC1 protein binding to *UNC5CL* mRNA in 786-O cells. **G-H** The effect of *YTHDC1* knockdown on UNC5CL expression in *ZC3H13* overexpressing cells was detected by qPCR and western blot. **I-J** Western blot analysis of IκBα, p-IκBα, p65 and p-p65 expression following *ZC3H13* knockdown and overexpression. **K** Nuclear and cytoplasmic fractionation assays were conducted to determine the subcellular distribution of p65 after *ZC3H13* knockdown. **L** Immunofluorescence analysis of p65 subcellular localization in the cytoplasm and nucleus of 786-O-shCtrl and 786-O-sh*ZC3H13* cells. **M-O** Effect of JSH-23 on sh*ZC3H13*-induced CSF2 expression and secretion in Renca and 786-O cells. **P** Effect of UNC5CL overexpression (oe*UNC5CL*) on sh*ZC3H13*-induced CSF2 expression and secretion. All histogram chart data are presented as the mean ± SD. Statistical analyses were performed by Graphpad Prism 9.5. ns, not significant, *P < 0.05, **P < 0.01

We next explored whether and how ZC3H13 affects UNC5CL expression in RCC. Given that m^6^A modifications have a significant impact on mRNA decay, we hypothesized that ZC3H13 could regulate UNC5CL mRNA stability. As anticipated, *ZC3H13* knockdown abolishes the stability of UNC5CL (Fig. 6A). whereas ZC3H13 overexpression exerted an opposite effect (Fig. 6B). To uncover the m^6^A “reader” proteins responsible for ZC3H13-dependent stabilization of UNC5CL, we systematically knockdown the expression of m^6^A readers (YTHDF1/2/3, YTHDC1/2, and IGF2BP1/2/3) in 786-O cells. Among them, only *YTHDC1* knockdown led to a significant reduction in both mRNA and protein level of UNC5CL in 786-O and ACHN cells (Fig. 6C-D, Supplementary Figs. 13 A-H). Furthermore, RNA stability assays revealed that UNC5CL mRNA half-life significantly decreased in *YTHDC1*-knockdown cells compared to controls (Fig. 6E). Consistently, RIP assay with anti-YTHDC1 antibody confirmed the direct interaction between YTHDC1 and *UNC5CL* mRNA in 786-O cells (Fig. 6F). Moreover, immunohistochemical staining revealed that the positive correlation between YTHDC1 and UNC5CL expression in clinical specimens (Supplementary Figs. 14). To further investigate whether YTHDC1 mediates ZC3H13-enhanced stability of *UNC5CL* mRNA, we knockdown *YTHDC1* in cells overexpressing *ZC3H13*. As shown in the Fig. 6G-H, overexpression of *ZC3H13* increased UNC5CL expression, whereas knockdown of *YTHDC1* reversed the positive regulatory effect of *ZC3H13* overexpression on UNC5CL. Therefore, these results indicate that YTHDC1 acts as a critical m^6^A reader protein responsible for mediating ZC3H13-dependent stabilization of *UNC5CL* mRNA.

### Loss of *ZC3H13* activates the NF-κB-CSF2 signaling axis via m^**6**^A modification of UNC5CL

UNC5CL is known to interact with subunit p65 of NF-κB, thereby suppressing NF-κB activation and NF-κB-dependent transcription by impairing the binding of NF-κB to its target sequences [22]. We hypothesized that sh*ZC3H13* may activate NF-κB signaling in RCC via downregulation of UNC5CL. As expected, we discovered that knockdown of *ZC3H13* upregulates the phosphorylation level of p65 at Ser536, whereas it exerted no significant effect on either the total or phosphorylated levels of IκBα. Conversely, *ZC3H13* overexpression similarly did not affect total and phosphorylated IκBα levels, but significantly decreased the expression of p-p65 (Fig. 6I-J, Supplementary Figs. 15 A). Immunofluorescence and nuclear-cytoplasmic fractionation assays further revealed that knockdown of *ZC3H13* markedly enhanced the nuclear translocation of p65 (Fig. 6K-L, Supplementary Figs. 15B). These findings collectively suggest that *ZC3H13* knockdown activates the NF-κB pathway independently of the classical IκBα-dependent mechanism.

Activated p65 has been reported to directly bind the CSF2 promoter and drive its transcription, we asked whether sh*ZC3H13* promotes CSF2 expression and secretion via NF-κB activation. Treatment with the NF-κB inhibitor JSH-23 reversed sh*ZC3H13*-induced phosphorylation of p65 as well as CSF2 expression and secretion, supporting the involvement of NF-κB in this process (Fig. 6M-O).

To further verify that UNC5CL acts as the molecular link between ZC3H13 and NF-κB signaling, we exogenously introduced UNC5CL in *ZC3H13*-knockdown cells. The results verify that *ZC3H13* knockdown led to increased p‑p65 and CSF2 expression, along with enhanced CSF2 secretion. Notably, UNC5CL overexpression reversed these increases without affecting IκBα or p‑IκBα levels, suggesting that UNC5CL specifically targets p65 activation independent of upstream IκBα regulation. Together, these data demonstrate that Loss of *ZC3H13* activates the NF-κB-CSF2 signaling axis via m^6^A modification of UNC5CL.

### CSF2 blockade in combination with anti-PD1 exerts stronger antitumor effects in RCC

**Fig. 7:**
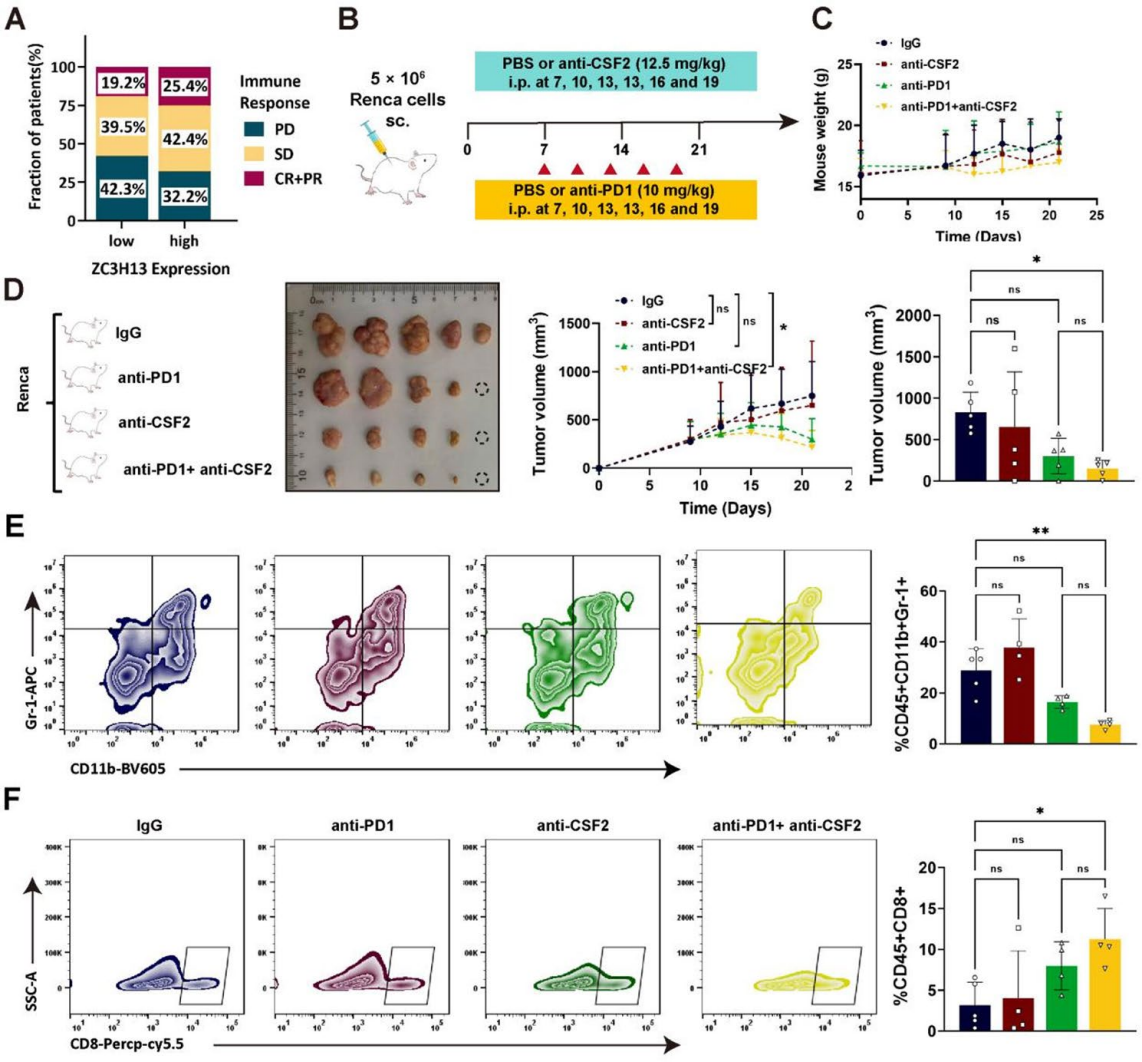
CSF2 blockade in combination with anti-PD1 exerts stronger antitumor effects in RCC. **A** The histogram shows the relationship between ZC3H13 expression level and immunotherapy response in the CheckMate 025 cohort. **B** Schematic of the experimental design for treating Renca-sh*Zc3h13* allograft-bearing mice with anti-CSF2 alone or in combination with anti-PD-1. **C** Body weights of mice across the four experimental groups (*n* = 5). **D** Representative photographs of resected tumors (left), and tumor growth curves across treatment groups (middle), quantitative measurements of tumor volume (right) at the endpoint of experienment (*n* = 5). **E-F** Flow cytometry analysis of tumor-infiltrating MDSCs and CD8^+^ T cells across the different treatment groups. All histogram chart data are presented as the mean ± SD. Statistical analyses were performed by Graphpad Prism 9.5. ns, not significant, *P < 0.05, **P < 0.01

We then asked whether ZC3H13 expression could act as a potential predictive value for immunotherapy. Among the RCC cohort receiving nivolumab in CheckMate 025, the patients with ZC3H13 high expression had better response to immunotherapy than those with ZC3H13 low expression. Specifically, the ZC3H13-high group achieved an objective response rate (ORR; CR + PR) of 25.4% and a clinical benefit rate (CBR; CR + PR + SD) of 67.8% (Fig. 7A).

Given that MDSCs inhibit responses to immunotherapy, we next asked whether the combination of anti-CSF2 and anti-PD1 would achieve better therapeutic efficacy in RCC. To this end, we established a subcutaneous tumor model using Renca cells which were treated with anti-CSF2 and/or PD-1 antibody for five cycles (Fig. 7B). As shown in Fig. 7D, neither anti-CSF2 nor anti-PD1 monotherapy significantly reduced tumor volume compared with the control group. In contrast, the combination treatment significantly inhibited tumor growth relative to the control group. Although the combination group showed lower tumor burden (149.3 mm^3^) than the anti-PD1 monotherapy burden (299 mm^3^), the difference did not reach statistical significance. These results demonstrated that the combination treatment showed combination of anti-CSF2 and anti-PD1 would achieve better therapeutic efficacy in RCC.

**Fig. 8:**
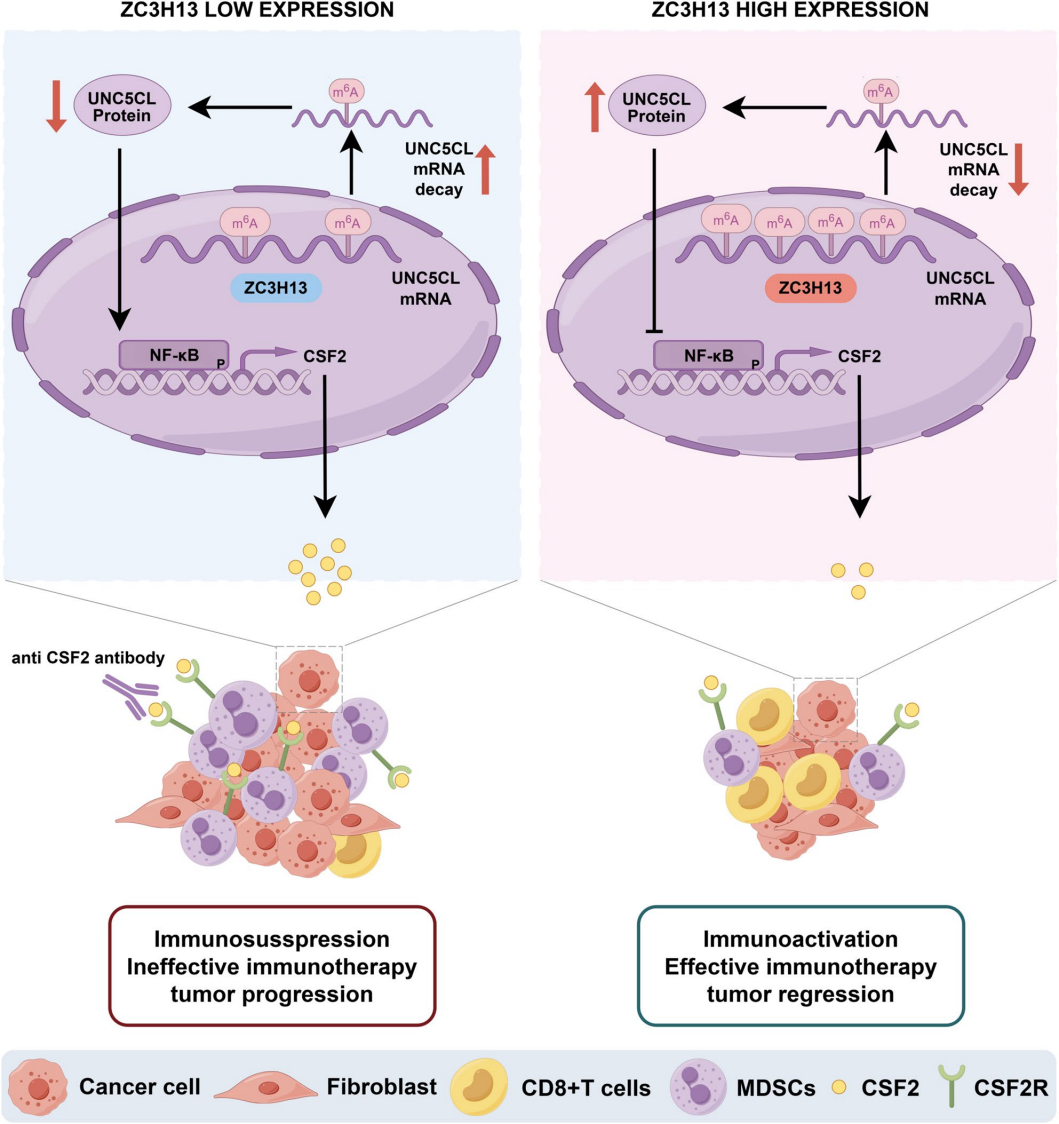
A schematic model illustrating our findings on ZC3H13-mediated m6A regulation

Furthermore, we explored whether the combination therapy reduces MDSCs infiltration in the tumor microenvironment, thereby facilitating the transition from “cold” to “hot” tumors. Flow cytometry data demonstrated that, compared with the control group, none of the monotherapy groups significantly reduced MDSCs infiltration; however, the combination therapy group exhibited a marked reduction in MDSCs infiltration (Fig. 7E). Flow cytometric analysis of tumor CD8^+^ T cells also showed that the combination group exhibited significantly elevated proportions of CD8^+^ T cells compared to control group (7 F). Taken together, these findings indicate that CSF2 blockade in combination with anti-PD1 exerts stronger antitumor effects in RCC by synergistically reversing the MDSCs mediated immunosuppressive microenvironment, providing a novel mechanistic basis for the observed therapeutic synergy in this mouse RCC model. A schematic model summarizing the key mechanisms in presented in Fig. 8.

## Discussion

m^6^A stands as the predominant internal modification of mRNA in mammals, orchestrating critical regulatory processes of mRNA splicing, translation, and degradation that are vital for gene expression homeostasis [23]. ZC3H13 serves as a pivotal component of the m^6^A methylation machinery, functioning within the writer complex alongside with METTL3, METTL14, WTAP, and VIRMA, and thereby facilitating m^6^A modification on RNA substrates. ZC3H13 regulates m^6^A modification by controlling the cellular localization of complex members WTAP, Virilizer, and Hakai. Inhibition of ZC3H13 expression leads to the translocation of WTAP, Virilizer, and Hakai proteins from the nucleus to the cytoplasm, accompanied by a reduction in the nuclear components of the methyltransferases METTL3 and METTL14, thereby suppressing m^6^A formation. Conversely, knockdown of WTAP, Virilizer, and Hakai does not affect the nuclear localization of ZC3H13 [24], suggesting its unique role in m^6^A modification. The exact contribution of ZC3H13 to tumorigenesis remains a contentious topic, with emerging literature presenting diverse roles across various malignancies, oscillating between tumor-promoting and tumor-suppressive potentials [18, 25–27]. This study unambiguously establishes ZC3H13 as a tumor suppressor in RCC, where its diminished expression correlates with poor patient prognosis. Beyond its direct implications for malignant cell properties, ZC3H13 is intricately involved in sculpting the tumor immune microenvironment in a context-sensitive manner. For instance, in breast cancer, elevated ZC3H13 levels correspond with enhanced infiltration of CD8^+^ T cells and dendritic cells (DCs), while negatively influencing regulatory T cell levels [19]. Conversely, in ovarian cancer presents an inverse relationship, where high ZC3H13 expression associates with reduced infiltration of these effector immune cells [20]. These dichotomous findings underscore the multifaceted role of ZC3H13 in modulating the tumor immune landscape, dependent on the cancer context.

Given the tumor-suppressive role of ZC3H13 in RCC, the mechanisms underlying its downregulation warrant careful consideration. Emerging evidence points to microRNAs as key negative regulators of ZC3H13 expression. Wu et al. showed that miR-362-3p and miR-425-5p directly target the ZC3H13 3′UTR and inhibit its expression in hepatocellular carcinoma [28], while Guo et al. found that exosomal miR-200c-3p reduces ZC3H13 expression in glioblastoma under hypoxic conditions [29]. Specifically, we have observed that miR-16-5p, which is upregulated in RCC and capable of targeting the ZC3H13 3′UTR, acts as an upstream repressor of ZC3H13 in RCC (our unpublished data). Beyond miRNA-mediated regulation, hypoxia itself may suppress ZC3H13 expression. Zhuang et al. observed markedly reduced ZC3H13 levels under hypoxic stress in patients with smoking-related coronary artery disease [30]. Notably, RCC is a prototypical hypoxia-associated tumor, characterized by constitutive activation of the VHL-HIF2α axis [31]. Therefore, sustained hypoxic stress may contribute to ZC3H13 downregulation in RCC, a possibility that warrants further investigation.

Despite the reported benefits of PD-1/PD-L1 blockade as a cornerstone of immunotherapy, results in RCC remain disappointingly suboptimal [32]. Paradoxically, even as RCC is regarded as an immunologically “hot” tumor with abundant CD8^+^ T cells, advanced RCC often exhibits poor response to immunotherapy, a paradox where high T cell infiltration correlates with unfavorable prognosis [5]. A key contributor to this phenomenon is the presence of MDSCs, whose accumulation within the TME profoundly impairs T and NK cell function, thereby facilitating tumor immune evasion [33, 34]. In our research, we demonstrate that ZC3H13 plays a crucial role in modulating TME composition by negatively regulating MDSCs accumulation. Our integrative analysis of both murine models and patient-derived clinical data substantiates an inverse correlation between ZC3H13 levels and MDSCs presence, reinforcing the concept that ZC3H13 serves as a suppressor of RCC progression by mitigating MDSC infiltration. In vitro coculture experiments further elucidate that the absence of ZC3H13 fosters the chemotaxis and conversion of MDSCs. MDSCs are abundant immunosuppressive cells in the RCC microenvironment. Growing evidence indicates that the abundance of MDSCs in the TME is critical for RCC progression and immunosuppression [9], and high levels of MDSCs infiltration in RCC tissues are significantly associated with shortened overall survival in patients [35]. These results strongly suggest that ZC3H13 may suppress RCC progression by reducing MDSCs infiltration in the TME.

The role of paracrine signaling in fostering MDSCs recruitment cannot be understated. Heterogeneous tumor cells create chemotactic gradients that not only mobilize MDSCs but elicit pro-inflammatory signaling cascades, notably involving STAT3 and NF-κB pathways that steer myeloid cells towards an immunosuppressive phenotype [36]. Our comprehensive analyses using RNA-sequencing and PCR arrays have pinpointed CSF2 (also known as GM-CSF) as a pivotal cytokine that is significantly upregulated upon *ZC3H13* loss. CSF2 is a cytokine that drives the expansion and activation of granulocytes, monocytes, macrophages, and dendritic cells in response to stress, infection, and cancer [37]. CSF2 plays a pivotal role in tumor immunoregulation, but it acts as a “double-edged sword” in cancer: it can restore neutrophil-mediated antitumor immunity, enhance DCs differentiation, and promote antitumor T-cell responses, yet it also promotes the expansion of immunosuppressive cells (e.g., MDSCs) and enhances EMT, angiogenesis, and the expression of immune checkpoint molecules, further supporting cancer progression [37]. Our findings indicate that CSF2 is not merely a bystander but an oncogenic factor in RCC, its levels markedly elevated in malignant tissues when compared to adjacent non-tumorous samples. Furthermore, therapeutic blockade of CSF2 effectively negated the pro-tumorigenic consequences of *ZC3H13* silencing.

Based on our findings, we propose that targeting CSF2 is a promising strategy for curbing RCC tumorigenesis. Our results reveal a synergistic advantage when combining CSF2 antagonism with anti-PD-1 therapy, signifying a potential therapeutic paradigm shift in RCC management. The bifunctional nature of CSF2 necessitates a nuanced approach, whereby strategies range from leveraging it as an immunogenic adjuvant to reverse immune suppression through anti-CSF2 interventions that decrease MDSC populations and promote productive T cell responses [38, 39]. The efficacy of such CSF2-centered modalities hinges upon clarifying its multifaceted roles, which are inherently tied to cancer type, underlying microenvironmental contexts, and treatment-specific parameters such as dosage and administration frequency [40].

We also observed notable cell line-specific discrepancies in our studies, particularly regarding the upregulation of CSF2 following ZC3H13 knockdown. Specifically, the 786-O cell line exhibited a pronounced response compared to the ACHN cell line. potentially correlating with their respective Hippel-Lindau (VHL) status, 786-O being VHL-deficient and ACHN retaining VHL function. Prior investigations by Zhang et al. have elucidated that VHL loss precipitates the ubiquitination and degradation of METTL3, leading to diminished global m^6^A levels [41]. This raises intriguing possibility of a regulatory interplay between VHL status and ZC3H13 in modulating CSF2 expression.

Integrated analysis of RNA-seq and MeRIP-seq identified UNC5CL as a potential target of ZC3H13. As UNC5CL has been characterized as a negative regulator of NF-κB signaling [22], these findings raised the possibility that loss of ZC3H13 may enhance NF-κB activity through suppression of UNC5CL. In the canonical NF-κB cascade, pathway activation is typically initiated by IκBα phosphorylation and degradation, thereby enabling p65 nuclear translocation and transcriptional activity. Interestingly, neither knockdown nor overexpression of *ZC3H13* altered the levels of IκBα or p-IκBα, suggesting that ZC3H13 regulates NF-κB activation independently of the classical IκBα-dependent mechanism. This notion is further supported by previous studies indicating that UNC5CL may suppress NF-κB signaling by modulating post-translational modification of p65, such as its phosphorylation, rather than by altering IκBα turnover. Consistent with this model, UNC5CL overexpression had no detectable effect on IκBα or p- IκBα expression, but effectively reversed the increase in p-p65 induced by *ZC3H13* knockdown. Together, these findings support the conclusion that ZC3H13 depletion activates NF-κB signaling, at least in part, through downregulation of UNC5CL and subsequent enhancement of p65 phosphorylation.

Nevertheless, certain limitations must be acknowledged in this study. The lack of data from dedicated clinical cohorts hinders the validation of ZC3H13 and CSF2 as biomarkers to predict responses to ICB in RCC. Furthermore, the absence of effective pharmacological agents that can upregulate ZC3H13 expression limits explorations into its therapeutic potential, particularly in conjunction with anti-PD-1 therapies. Additionally, in our initial screening, we focused only on methyltransferases member, whereas m^6^A demethylases (“erasers”) such as FTO and ALKBH5 also play critical roles in modulating m^6^A modification levels and downstream biological functions. Moreover, significant mechanistic gaps remain to be addressed in this study, as we did not perform Co-IP/GST pull-down to verify the reduced UNC5CL-p65 interaction, nor ChIP-qPCR to assess p65 enrichment on the CSF2 promoter upon *ZC3H13* knockdown, leaving the proposed mechanistic link incomplete.

In conclusion, our findings elucidate the role and regulatory mechanisms by which ZC3H13, an RNA m^6^A methyltransferase, shapes an immunosuppressive microenvironment in RCC. Loss of ZC3H13 activates the NF-κB-CSF2 signaling axis through m^6^A modification of UNC5CL, driving MDSCs-mediated immune suppression and promoting tumor progression. Importantly, our data suggest that targeting CSF2, especially in combination with anti-PD-1 treatment, presents a novel therapeutic strategy with significant translational potential for patients with RCC.

## Conclusions

Ultimately, we identify that *ZC3H13* deficiency epigenetically activates CSF2 signaling, fostering an MDSCs-enriched niche that promotes tumor progression and confers resistance to immunotherapy. Furthermore, we provide evidence that a combination of anti-CSF2 and anti-PD1 treatments yields superior therapeutic efficacy, revealing a rational and synergistic treatment paradigm with significant translational potential.

## Supplementary Information

Supplementary Material 1.

## Data Availability

The datasets used and/or analysed during the current study are available from the corresponding author on reasonable request.

## Abbreviations

RCC: Renal cell carcinoma
KIRC: Kidney renal clear cell carcinoma
ICB: Immune checkpoint blockade
TME: Tumor immune microenvironment
MDSCs: Myeloid-derived suppressor cells
BMDCs: Bone marrow-derived cells
m^6^A: N6-methyladenosine
ZC3H13: Zinc finger CCCH-type containing 13
DCs: Dendritic cells
VHL: Von Hippel-Lindau
PLS-DA: Partial least squares discriminant analysis
TCGA: The cancer genome atlas
CFSE: Carboxyfluorescein succinimidyl ester
GM-CSF: granulocyte-macrophage colony stimulating factor
CSF2: colony stimulating factor 2
RIP: RNA immunoprecipitation
MeRIP-seq: m^6^A-modified RNA immunoprecipitation sequencing
RNA-seq: RNA sequencing
CCK-8: Cell counting kit-8
IHC: Immunohistochemistry
ELISA: Enzyme-Linked Immunosorbent Assay
FBS: Fetal Bovine Serum
GSEA: Gene Set Enrichment Analysis
KEGG: Kyoto Encyclopedia of Genes and Genomes
UNC5CL: UNC-5 Family C-Terminal Like
