# Supplementary material for: Epitranscriptomic silencing of the ZC3H13/m6A axis orchestrates immunosuppressive microenvironment remodeling in renal cell carcinoma via CSF2-mediated MDSCs recruitment
Source: J Exp Clin Cancer Res. 2026 May 20;45:133. doi: 10.1186/s13046-026-03742-2 (PMC13255311; doi:10.1186/s13046-026-03742-2)
Supplement: Supplementary file 1 — Supplementary Material 1. [file 13046_2026_3742_MOESM1_ESM.docx]

**Supplementay figure:**

**Supplementary Figure 1:**

**
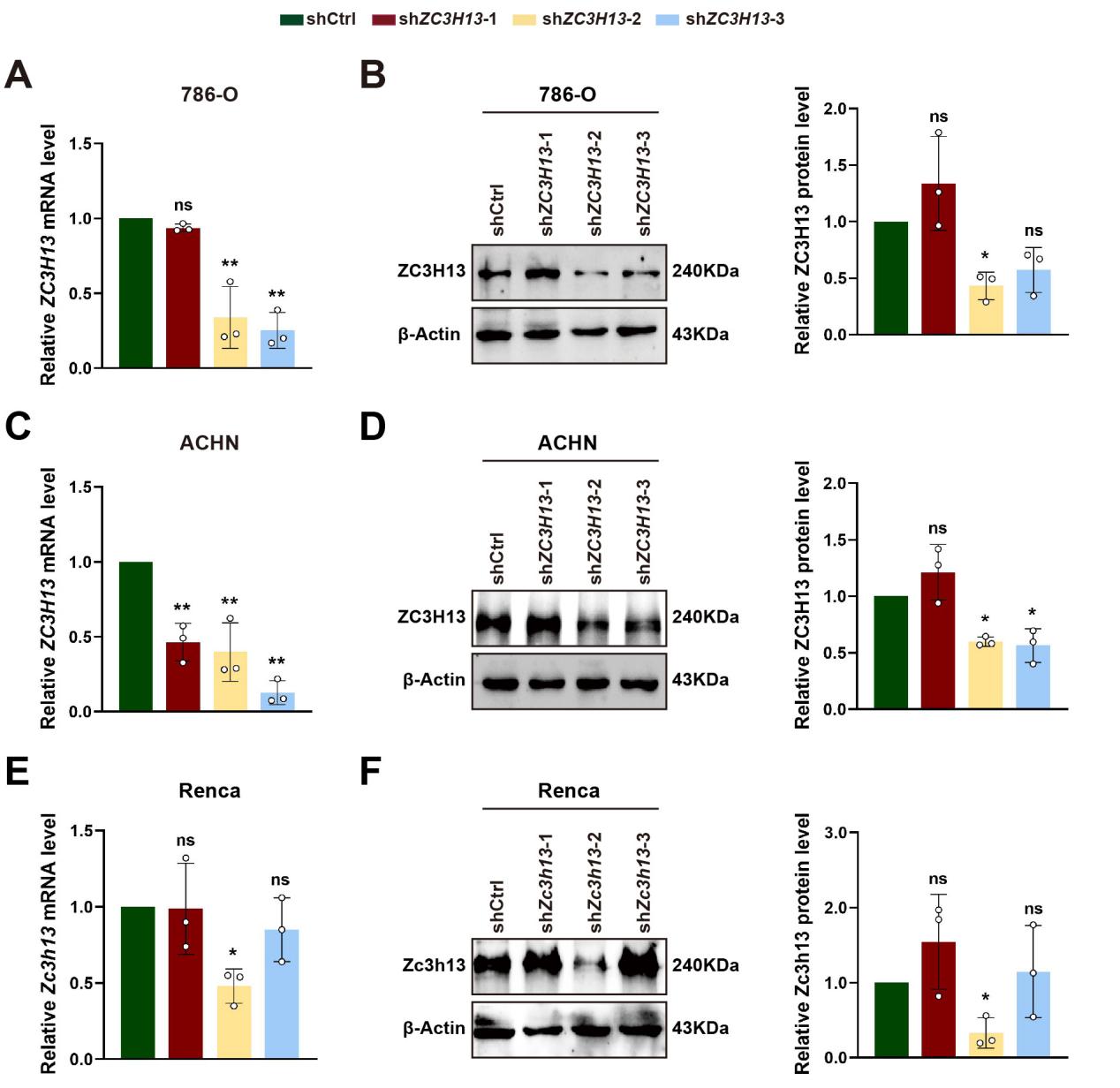
**

**Supplementary Figure 1: Validation of *ZC3H13* knockdown efficiency in RCC cell lines.** (A-B) Knockdown efficiency of *ZC3H13* was verified by qPCR and western blot in 786-O cells; (C-D) *ZC3H13* knockdown efficiency was verified by qPCR and western blot in ACHN cells; (E-F) Knockdown efficiency of *ZC3H13* was verified by qPCR and western blot in Renca cells. All histogram chart data are presented as the mean ± SD. Statistical analyses were performed by Graphpad Prism 9.5. ns, not significant, **P* < 0.05, ***P* < 0.01.

**Supplementary Figure 2:**

**
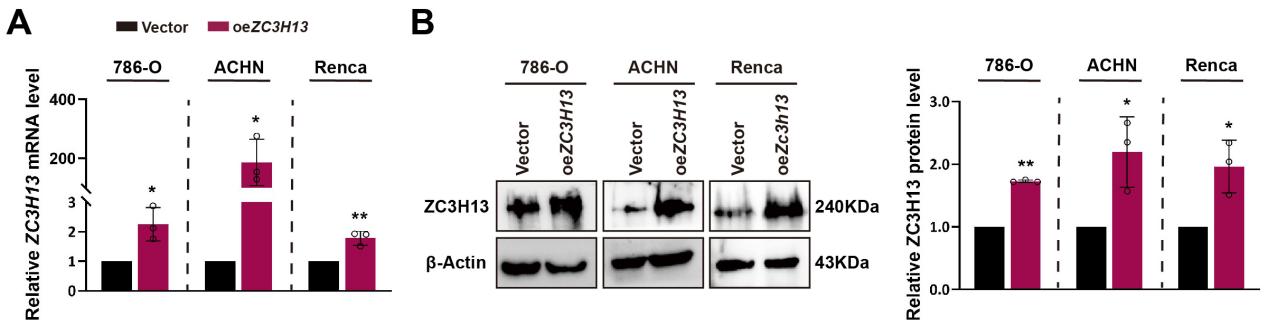
**

**Supplementary Figure 2: Validation of *ZC3H13* overexpression efficiency via qPCR and western blot in RCC cell lines.** All histogram chart data are presented as the mean ± SD. Statistical analyses were performed by Graphpad Prism 9.5. ns, not significant, **P* < 0.05, ***P* < 0.01.

**Supplementary Figure 3:**

**
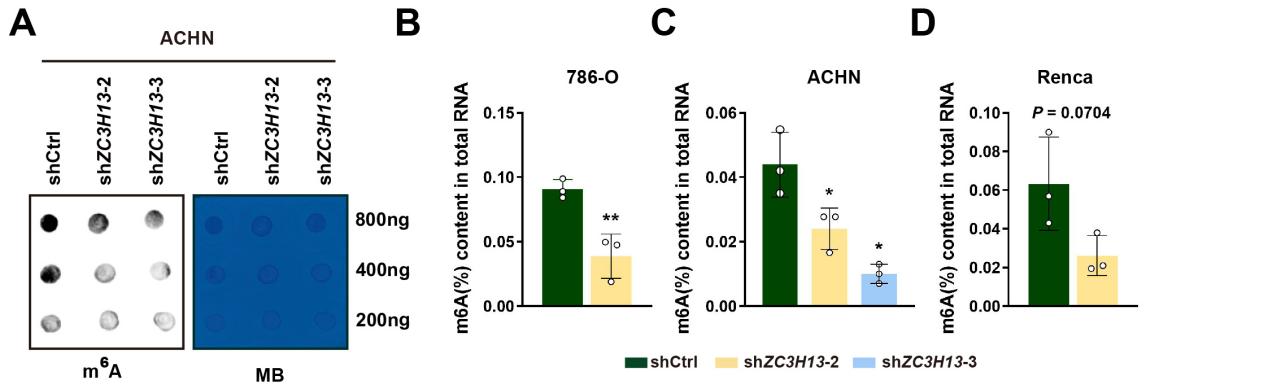
**

**Supplementary Figure 3: Global m^6^A modification levels are altered by ZC3H13 manipulation.** (A) Effects of *ZC3H13* knockdown on m^6^A modification levels in ACHN cells, as determined by dot blot analysis. (B-D) Effects of *ZC3H13* knockdown on m^6^A modification levels in 786-O/ACHN/Renca cells, as measured by colorimetric ELISA-like assay via the m^6^A RNA methylation quantification kit. All histogram chart data are presented as the mean ± SD. Statistical analyses were performed by Graphpad Prism 9.5. ns, not significant, **P* < 0.05, ***P* < 0.01.

**Supplementary Figure 4:**


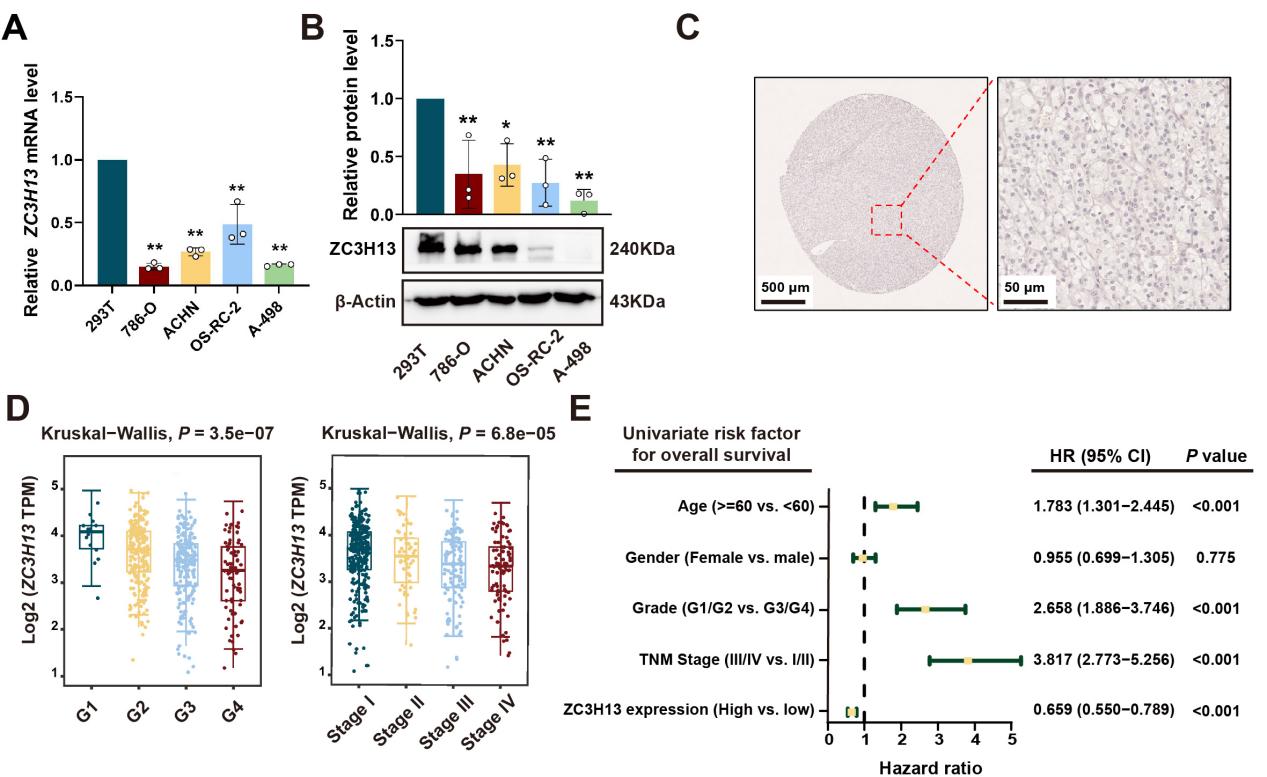


**Supplementary Figure 4: ZC3H13 Expression in RCC cell lines and its correlation with pathological grade, stage, and prognosis.** (A-B) ZC3H13 expression in 293T and human RCC cell lines (786-O, ACHN, OS-RC-2, A-498) was measured by qPCR and western blot. (C) Immunostaining with isotype antibody. (D-E) Box plots showing ZC3H13 expression across pathological grades and stages. (F) Univariate Cox regression analysis of overall survival in the TCGA-KIRC cohort. Forest plot showing the hazard ratios (HR) and 95% confidence intervals (CI) for each clinical variable, including age (≥60 vs. <60), gender (female vs. male), grade (G1/G2 vs. G3/G4), TNM stage (III/IV vs. I/II), and ZC3H13 expression (high vs. low, stratified by the median expression level of ZC3H13). All histogram chart data are presented as the mean ± SD. Statistical analyses were performed by Graphpad Prism 9.5. ns, not significant, **P* < 0.05, ***P* < 0.01.

**Supplementary Figure 5:**


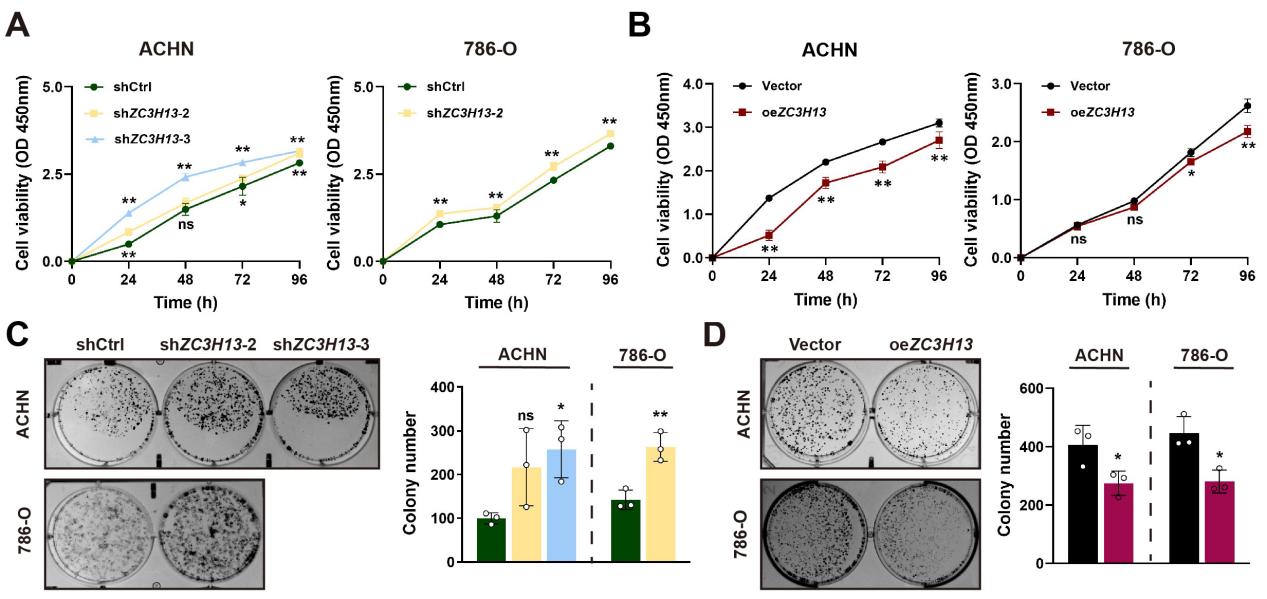


**Supplementary Figure 5: ZC3H13 negatively regulates RCC cell proliferation.** (A, C) Knockdown of *ZC3H13* enhanced cell proliferation as assessed by CCK-8 assay (A) and colony formation assay (C). (B, D) Overexpression of *ZC3H13* attenuated cell proliferation, evaluated by CCK-8 assay (B) and colony formation assay (D). All histogram chart data are presented as the mean ± SD. Statistical analyses were performed by Graphpad Prism 9.5. ns, not significant, **P* < 0.05, ***P* < 0.01.

**Supplementary Figure 6:**

**
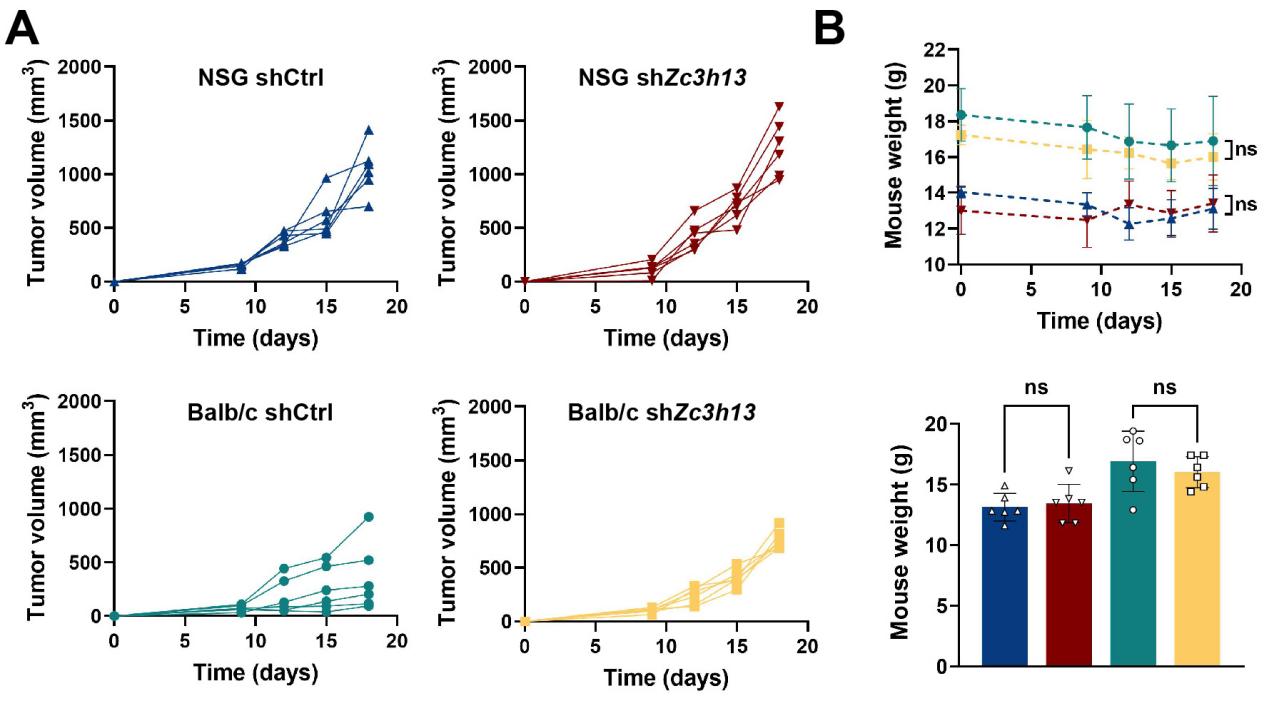
**

**Supplementary Figure 6: Effects of *Zc3h13* knockdown on tumor growth and body weight in Balb/c and NSG mice.** (A) Tumor growth curves of the four experimental groups (n = 6). (B) Mouse weight variations over time (top) and final mouse weight at the experimental endpoint (bottom panel) across the four experimental groups. All histogram chart data are presented as the mean ± SD. Statistical analyses were performed by Graphpad Prism 9.5. ns, not significant, **P* < 0.05, ***P* < 0.01.

**Supplementary Figure 7:**

**
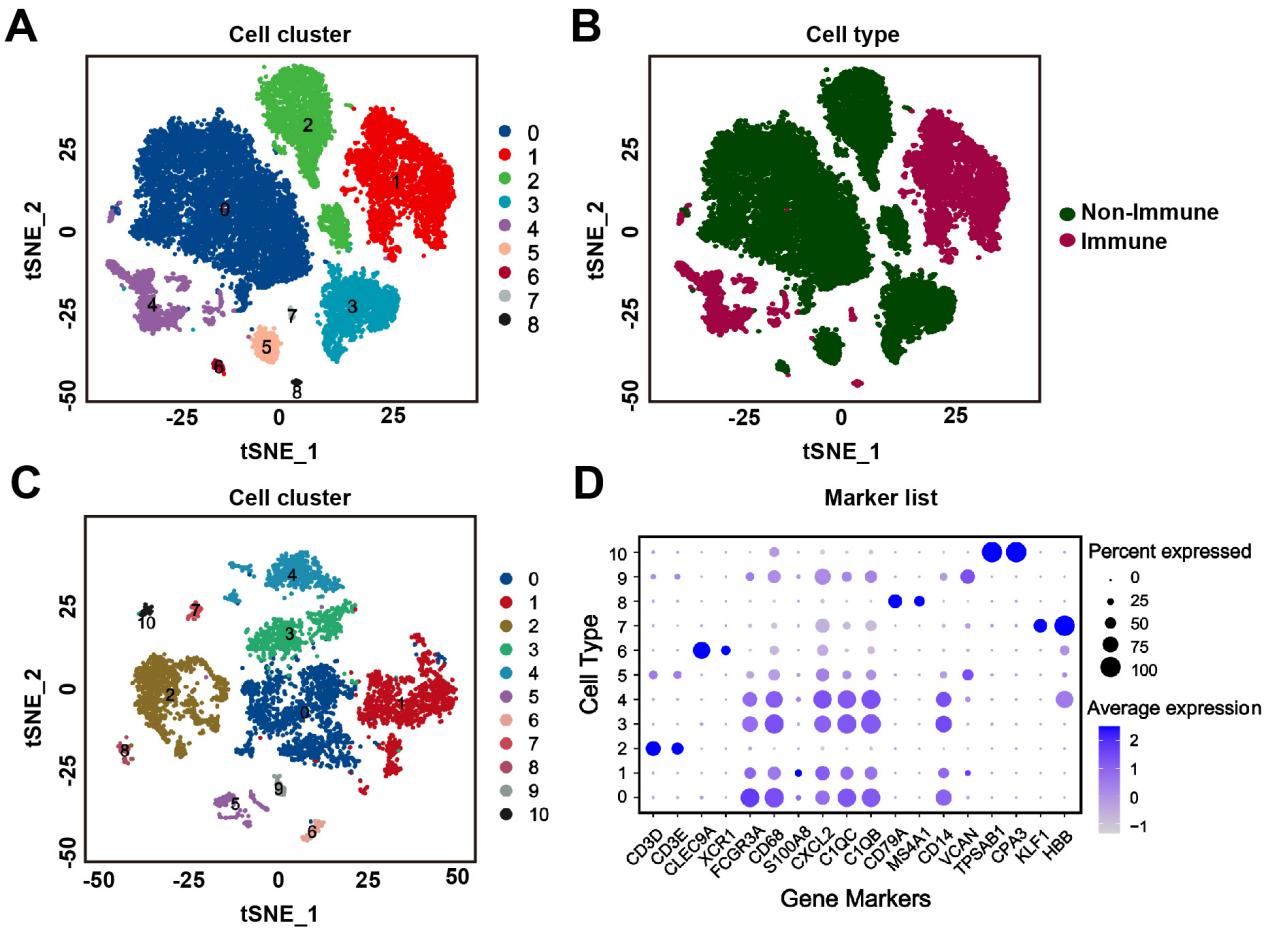
**

**Supplementary Figure 7: Overview of Single Cells Derived from GSE159115.** (A-B) tSNE plot of all the single cells, with each color coded for (A) 9 major cell types, and (B) immune cell (CD45+ ) or non-immune cell (CD45-). (C) tSNE plot of immune cells, with each color coded for 10 major cell types. (D) Top two marker genes of 10 major cell types identified in this profile.

**Supplementary Figure 8:**


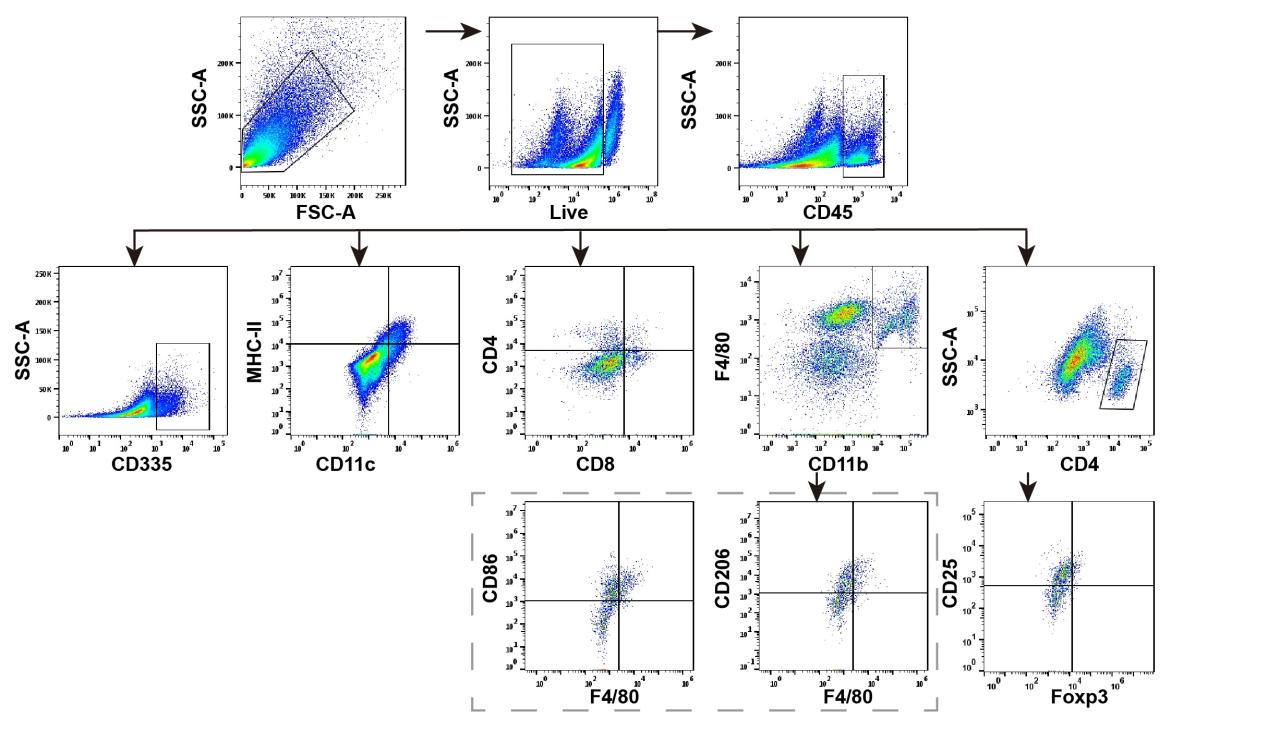


**Supplementary Figure 8: Comprehensive profiling of immune cell infiltration in allograft tumors.** Gating strategy of tumor infiltrating immune cells, including Helper T cell (CD45^+^CD4^+^), Cytotoxic T cell (CD45^+^CD8^+^), NK cell (CD45^+^CD335^+^), MDSCs (CD45^+^CD11b^+^Gr-1^+^), dendritic cell (CD45^+^CD11c^+^MHCII^+^), M1 macrophage (CD45^+^CD11b^+^F4/80^+^CD86^+^), M2 macrophage (CD45^+^CD11b^+^F4/80^+^CD206^+^) and regulatory T cells (Tregs, CD45^+^CD4^+^CD25^+^Foxp3^+^).

**Supplementary Figure 9:**


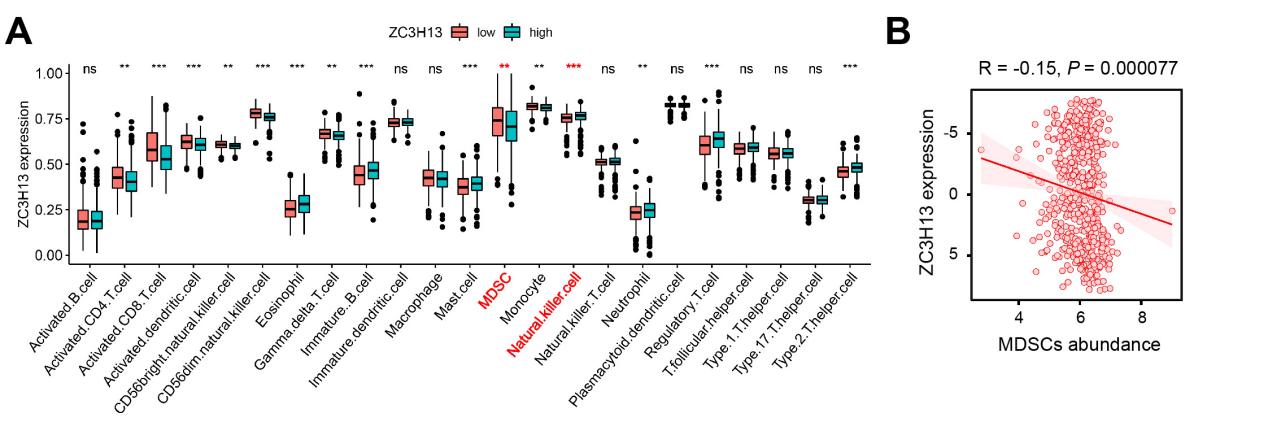
**Supplementary Figure 9: Low ZC3H13 expression group exhibited increased infiltration of MDSCs.** (A) ssGSEA assessing the relationship between ZC3H13 mRNA expression and the infiltration levels of 23 immune cell types in the renal cancer immune microenvironment; (B) Analysis of the correlation between ZC3H13 expression and MDSCs infiltration in renal cell carcinoma using the TSIDB database. ns, not significant, **P* < 0.05, ***P* < 0.01, ****P* < 0.001.

**Supplementary Figure 10:**


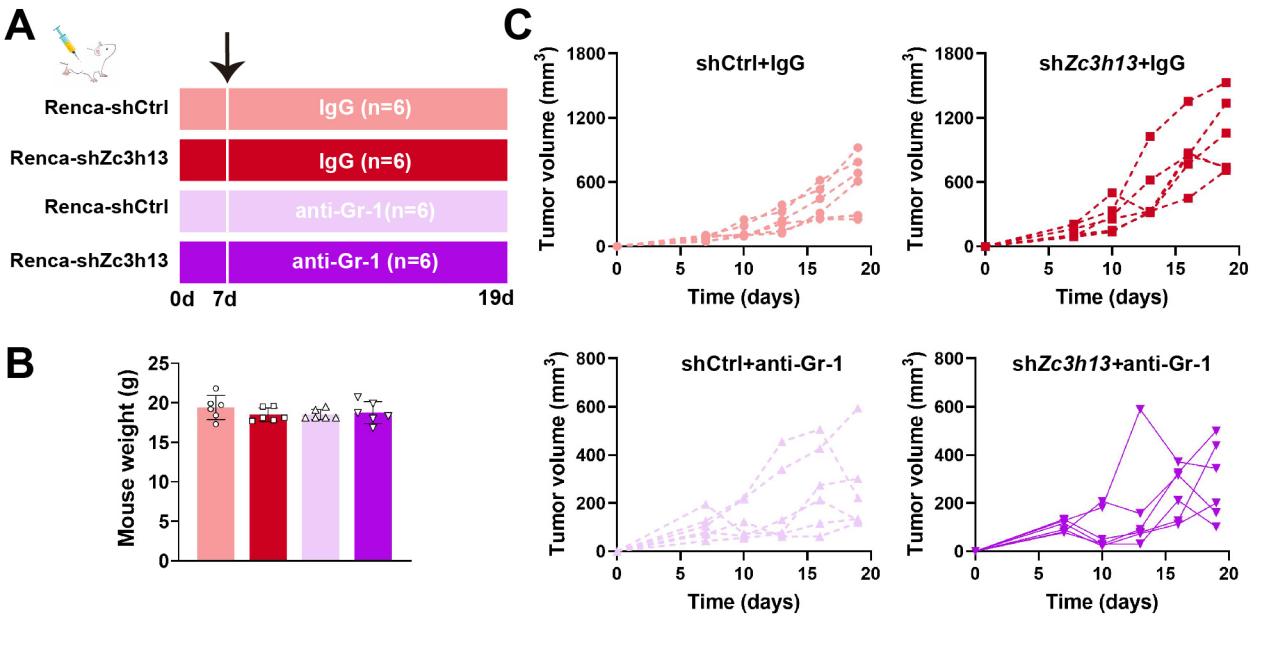


**Supplementary Figure 10:** (A) Schematic model depicting the experimental design of anti-Gr-1 treatment or IgG in Renca-shCtrl and Renca-sh*Zc3h13* allograft-bearing mice. (B) Body weights of mice across the four experimental groups. (C) Tumor growth curves of the four experimental groups.

**Supplementary Figure 11:**


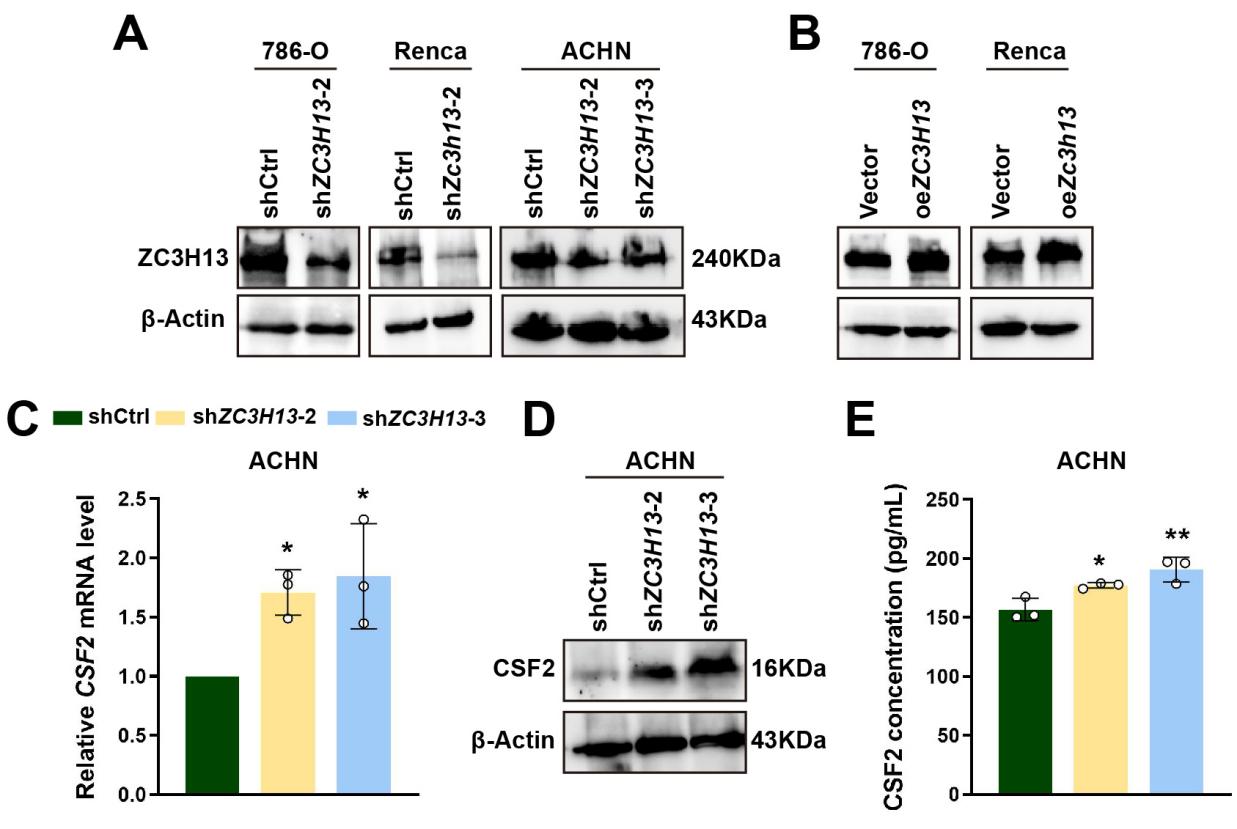


**Supplementary Figure 11: Knockdown of *ZC3H13* promotes CSF2 expression and secretion in ACHN cells.**

1. The knockdown efficiency of ZC3H13 in 786-O, ACHN, and Renca cells was demonstrated on 7.5% separating gel. (C-D) The mRNA and protein expression levels of CSF2 following *ZC3H13* knockdown in ACHN cells were detected by qPCR and western blot. (C) ELISA analysis of CSF2 secretion in the supernatant of ACHN cells with or without *ZC3H13* knockdown. All histogram chart data are presented as the mean ± SD. Statistical analyses were performed by Graphpad Prism 9.5. ns, not significant, **P* < 0.05, ***P* < 0.01.

**Supplementary Figure 12:**


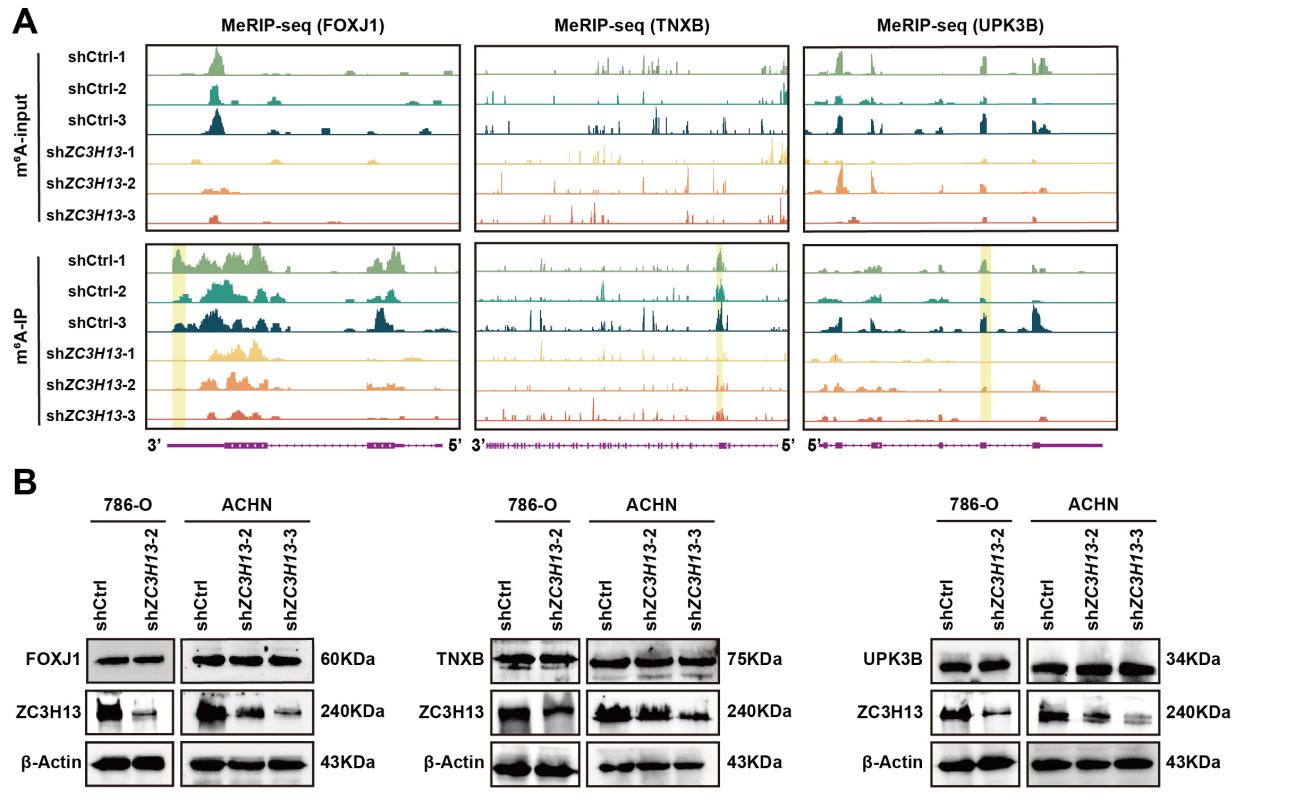


**Supplementary Figure 12: Screening and validation of downstream targets of ZC3H13.** (A) IGV visualization of MeRIP-seq reads along indicated mRNAs. (B) Protein levels of TNXB, UPK3B, and FOXJ1 following *ZC3H13* knockdown in 786-O/ACHN cells.

**Supplementary Figure 13:**


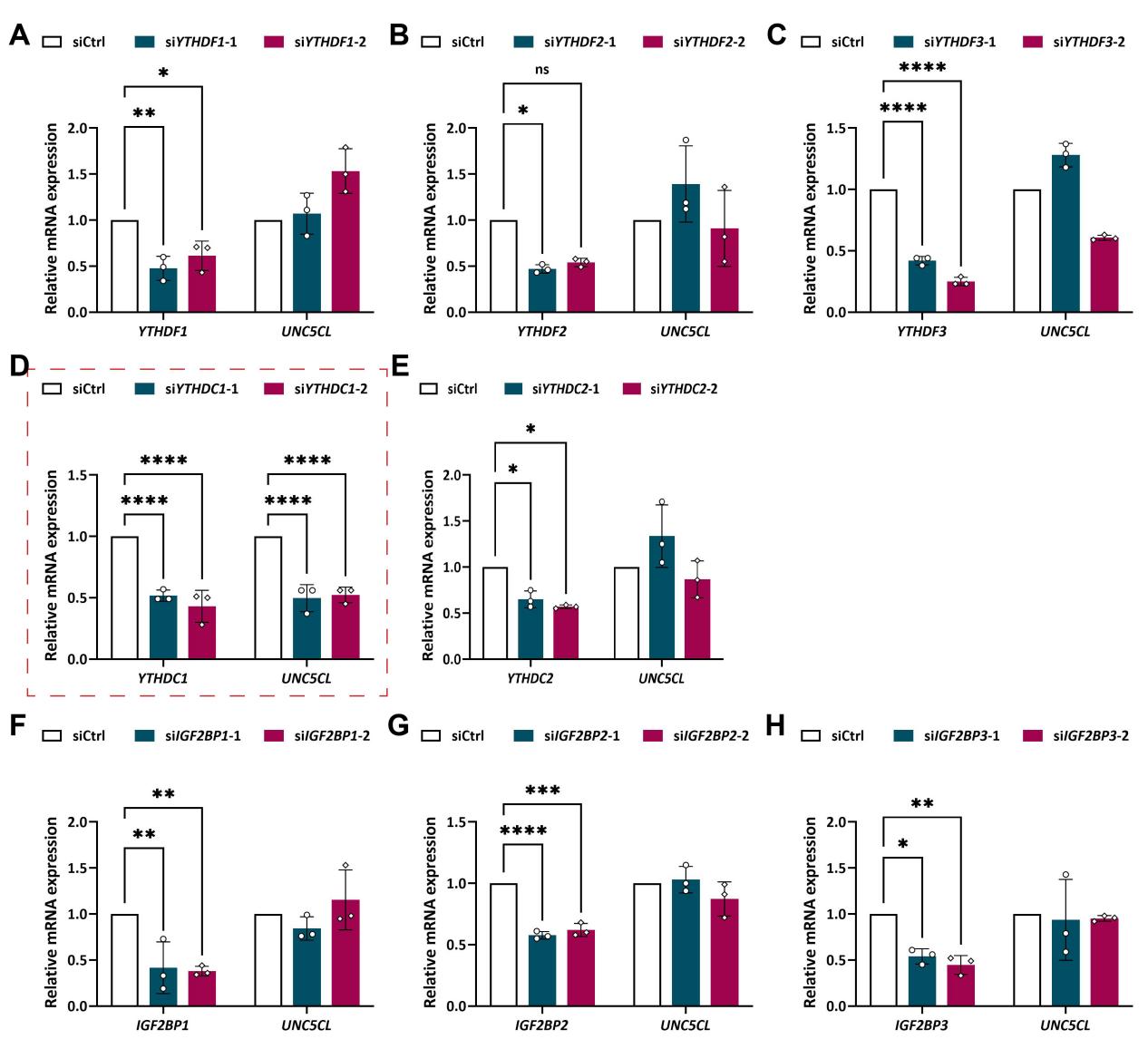


**Supplementary Figure 13: YTHDC1-mediated stabilization of UNC5CL transcripts.** (A-C) Effect of m^6^A reader (YTHDF1/2/3) silencing on *UNC5CL* mRNA level in 786-O cells. (D-E) Effect of m^6^A reader (YTHDC1/2) silencing on *UNC5CL* mRNA level in 786-O cells. (F-H) Effect of m^6^A reader (IGF2BP1/2/3) silencing on *UNC5CL* mRNA levels in 786-O cells. All histogram chart data are presented as the mean ± SD. Statistical analyses were performed by Graphpad Prism 9.5. ns, not significant, **P* < 0.05, ***P* < 0.01.

**Supplementary Figure 14:**


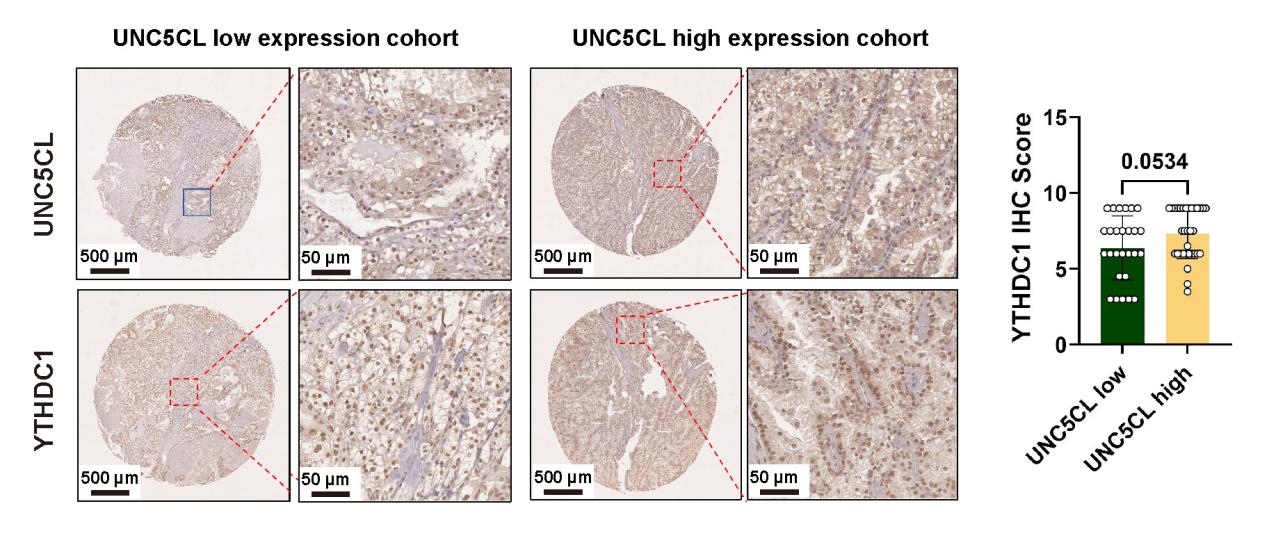


**Supplementary Figure 14: YTHDC1 and UNC5CL exhibit a positive correlation.** Representative immunohistochemical (IHC) images of UNC5CL and YTHDC1 in human RCC tissue microarrays (left), along with Spearman’s correlation analysis of their expression levels (right). All histogram chart data are presented as the mean ± SD. Statistical analyses were performed by Graphpad Prism 9.5. ns, not significant, **P* < 0.05, ***P* < 0.01.

**Supplementary Figure 15:**


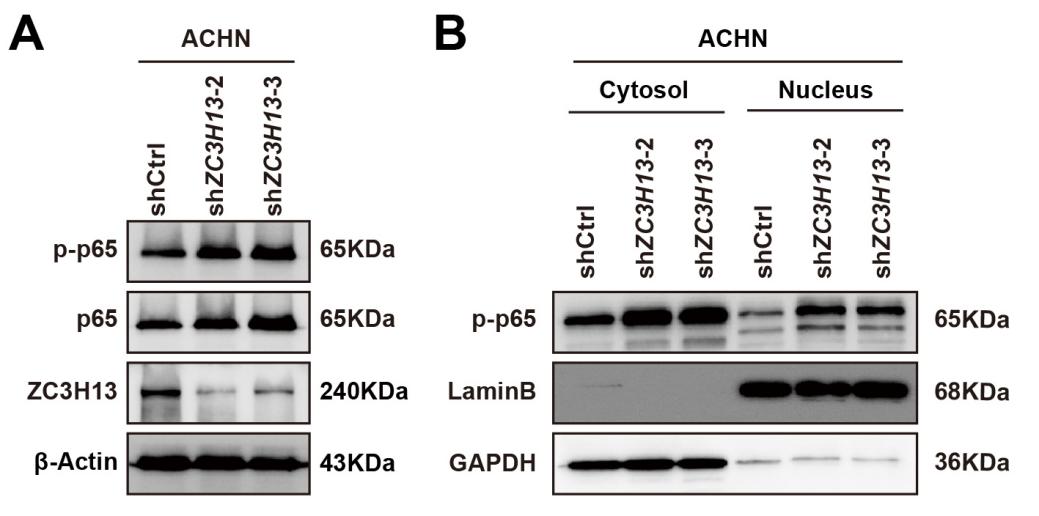


**Supplementary Figure 15: Loss of *ZC3H13* activates the NF-κB signaling axis.** (A) Western blot analysis of p65 and p-p65 expression following *ZC3H13* knockdown in ACHN cells. (B) Nuclear and cytoplasmic fractionation assays were conducted to determine the subcellular distribution of p-p65 after *ZC3H13* knockdown.

**Supplementary tables**

**Table S1. List of 30 m^6^A regulatory genes**

| Gene Symbol | Full Name | Classification |
| --- | --- | --- |
| ZC3H13 | Zinc Finger CCCH Domain-Containing Protein 13 | Writers |
| METTL3 | Methyltransferase 3 | Writers |
| METTL14 | Methyltransferase 14 | Writers |
| METTL16 | Methyltransferase 16 | Writers |
| WTAP | WT1 Associated Protein | Writers |
| RBM15 | RNA Binding Motif Protein 15 | Writers |
| RBM15B | RNA Binding Motif Protein 15B | Writers |
| VIRMA | Vir Like m6A Methyltransferase Associated | Writers |
| CBLL1 | Cbl Proto-Oncogene Like 1 | Writers |
| FTO | FTO Alpha-Ketoglutarate Dependent Dioxygenase | Erasers |
| ALKBH5 | AlkB Homolog 5, RNA Demethylase | Erasers |
| YTHDF1 | YTH N6-Methyladenosine RNA Binding Protein F1 | Readers |
| YTHDF2 | YTH N6-Methyladenosine RNA Binding Protein F2 | Readers |
| YTHDF3 | YTH N6-Methyladenosine RNA Binding Protein F3 | Readers |
| YTHDC1 | YTH N6-Methyladenosine RNA Binding Protein C1 | Readers |
| YTHDC2 | YTH N6-Methyladenosine RNA Binding Protein C2 | Readers |
| IGF2BP1 | Insulin Like Growth Factor 2 MRNA Binding Protein 1 | Readers |
| IGF2BP2 | Insulin Like Growth Factor 2 MRNA Binding Protein 2 | Readers |
| IGF2BP3 | Insulin Like Growth Factor 2 MRNA Binding Protein 3 | Readers |
| RBMX | RNA Binding Motif Protein X-Linked | Readers |
| ZNF217 | Zinc Finger Protein 217 | Readers |
| EIF3A | Eukaryotic Translation Initiation Factor 3 Subunit A | Readers |
| HNRNPA2B1 | Heterogeneous Nuclear Ribonucleoprotein A2/B1 | Readers |
| HNRNPCL1 | Heterogeneous Nuclear Ribonucleoprotein C Like 1 | Readers |
| FMR1 | Fragile X Messenger Ribonucleoprotein 1 | Readers |
| ABCF1 | ATP Binding Cassette Subfamily F Member 1 | Readers |
| ELAVL1 | ELAV Like RNA Binding Protein 1 | Readers |
| G3BP1 | G3BP Stress Granule Assembly Factor 1 | Readers |
| G3BP2 | G3BP Stress Granule Assembly Factor 2 | Readers |
| PRRC2A | Proline Rich Coiled-Coil 2A | Readers |

**Table S2. Target sequences of siRNAs**

|  | sense（5'-3'） | antisense（5'-3'） |
| --- | --- | --- |
| si*YTHDF1*-1 | GGCUGGAGAAUAACGACAATT | UUGUCGUUAUUCUCCAGCCTT |
| si*YTHDF1*-2 | GGAUACAGUUCAUGACAAUTT | AUUGUCAUGAACUGUAUCCTT |
| si*YTHDF2*-1 | GGUGGAUGGUAAUGGAGUATT | UACUCCAUUACCAUCCACCTT |
| si*YTHDF2*-2 | GCUCUGGAUAUAGUAGCAATT | UUGCUACUAUAUCCAGAGCTT |
| si*YTHDF3*-1 | CAUACAUCGUUCCAUUAAATT | UUUAAUGGAACGAUGUAUGTT |
| si*YTHDF3*-2 | GUCAGUGCUUCACCUUCUATT | UAGAAGGUGAAGCACUGACTT |
| si*YTHDC1*-1 | GCUCUGCAUCAGAGUCAUATT | UAUGACUCUGAUGCAGAGCTT |
| si*YTHDC1*-2 | GCAAGGAGUGUUAUCUUAATT | UUAAGAUAACACUCCUUGCTT |
| si*YTHDC2*-1 | GGCAGAGAACUGUUCUAAATT | UUUAGAACAGUUCUCUGCCTT |
| si*YTHDC2*-2 | GCAUCCUGUUUGAUGACAATT | UUGUCAUCAAACAGGAUGCTT |
| si*IGF2BP1*-1 | GAGAUGGUGCAGGUGUUUATT | UAAACACCUGCACCAUCUCTT |
| si*IGF2BP1*-2 | CGGUGAACGAGUUGCAGAATT | UUCUGCAACUCGUUCACCGTT |
| si*IGF2BP2*-1 | CAGUUUGAGAACUACUCCUTT | AGGAGUAGUUCUCAAACUGTT |
| si*IGF2BP2*-2 | GUGAAUCUCUUCAUCCCAATT | UUGGGAUGAAGAGAUUCACTT |
| si*IGF2BP3*-1 | GCUGAGAAGUCGAUUACUATT | UAGUAAUCGACUUCUCAGCTT |
| si*IGF2BP3*-2 | GCUAGACAAGCACUAGACATT | UGUCUAGUGCUUGUCUAGCTT |

**Table S3. Target sequences of shRNAs**

|  | sense（5'-3'） | antisense（5'-3'） |
| --- | --- | --- |
| sh*ZC3H13*-1 | CCTCACAATCAGGATCATCTA | TAGATGATCCTGATTGTGAGG |
| sh*ZC3H13*-2 | GAAAGTTCTCGTACGGAAATA | TATTTCCGTACGAAGACTTTC |
| sh*ZC3H13*-3 | CCTCTCCTTATCCTTCACATT | AATGTGAAGGATAAGGAGAGG |
| sh*Zc3h13*-1 | GCAGCTGCCTCTATGGAAACACTC | GAGTGTTTCCATAGAGGCAGCTGC |
| sh*Zc3h13*-2 | GATTCTGACAATGGAGATATTCTC | GAGAATATCTCCATTGTCAGAATC |
| sh*Zc3h13*-3 | GCTAGGGATGCTCGGGATATTCTC | GAGAATATCCCGAGCATCCCTAGC |

**Table S4. Primer sequences**

| **Quantitative real-time PCR** | |
| --- | --- |
| *GAPDH-*F | AGAAGGCTGGGGCTCATTTG |
| *GAPDH-*R | AGGGGCCATCCACAGTCTTC |
| *Gapdh-*F | AGGTCGGTGTGAACGGATTTG |
| *Gpadh-*R | GGGGTCGTTGATGGCAACA |
| *ZC3H13*-F | TCTGATAGCACATCCCGAAGA |
| *ZC3H13*-R | CAGCCAGTTACGGCACTGT |
| *Zc3h13-*F | GATGACCAGGGTAGTCGGAAG |
| *Zc3h13-*R | GGTTCTCCACGCTTTAACTGA |
| *CSF2*-F | CTGGAGCTGTACAAGCAGGG |
| *CSF2*-R | CACAGGAAGTTTCCGGGGTT |
| *Csf2*-F | GGGTCTACGGGGCAATTTCA |
| *Csf2*-R | CACAGTCCGTTTCCGGAGTT |
| *Nos2*-F | GTTCTCAGCCCAACAATACAAGA |
| *Nos2*-R | GTGGACGGGTCGATGTCAC |
| *Arg1*-F | CTCCAAGCCAAAGTCCTTAGAG |
| *Arg1*-R | AGGAGCTGTCATTAGGGACATC |
| *Cd274*-F | GCTCCAAAGGACTTGTACGTG |
| *Cd274*-R | TGATCTGAAGGGCAGCATTTC |
| *Asns*-F | GCAGTGTCTGAGTGCGATGAA |
| *Asns*-R | TCTTATCGGCTGCATTCCAAAC |
| *UNC5CL*-F | CGGAGAAAAGCAGCCGATGA |
| *UNC5CL*-R | ATCCTGGAAGGTGTGCATGG |
| *Unc5cl*-F | AAGGCAGCCAATGGGAATGA |
| *Unc5cl* -R | CAGGTCTCTTCCTCCGATGC |
| *FOXJ1-*F | GCCTCCCTACTCGTATGCCA |
| *FOXJ1-*R | GCCGACAGGGTGATCTTGG |
| *TNXB*-F | CCAAGACCATCACCACCATGA |
| *TNXB*-R | GTTGTCGGTGTCACAGCCA |
| *UPK3B*-F | CACTCTCCACCAAGGGAAGAC |
| *UPK3B*-R | ATGACGATCATGCTGCCACT |
| *YTHDF1*-F | TGGACACCCAGAGAACAAAAGG |
| *YTHDF1*-R | CCAATGGACGGCGGGTAATA |
| *YTHDF2*-F | CTGTTGGTAGCGGGTCCATT |
| *YTHDF2*-R | GCCCAAGATGCTGGTTTTGG |
| *YTHDF3*-F | TGTGGGAATTGGGGGTTCTG |
| *YTHDF3*-R | GGAGCCTTTACCACTGACCC |
| *YTHDC1*-F | TATGGTCCACGCTCCCTGTA |
| *YTHDC1*-R | GCTGGAAGCACCCAGTGTAT |
| *YTHDC2*-F | AAAAGAGTCAGGACTGGGGC |
| *YTHDC2*-R | ACGTTCCCATAACTGGAGCA |
| *IGF2BP1*-F | ATCGGCAACCTCAACGAGAG |
| *IGF2BP1*-R | GTTTCGATGGCCTTCATCGC |
| *IGF2BP2*-F | CTACGCCTTCGTGGACTACC |
| *IGF2BP2*-R | TGTTGACTTGTTCCACATTCTCC |
| *IGF2BP3*-F | ACTGCACGGGAAACCCATAG |
| *IGF2BP3*-R | TCCCACTGTAAATGAGGCGG |
| **MeRIP-qPCR** | |
| *UNC5CL*-F | CTGCCGTTCTGCTCCTCAAA |
| *UNC5CL*-R | GCTCCTACACCCTTCTCTTCC |
| *FOXJ1*-F | CGCTTCTTGGTCCCAGTAGTT |
| *FOXJ1*-R | AGTGTAGCTGTAGCTGAGGC |
| *UPK3B*-F | TCCTACTCTTGGCCTTCTTGG |
| *UPK3B*-R | ACTGGGACCAATTCAACAGAGG |
| *TNXB*-F | GCAATCGGTTCCAGTGTACC |
| *TNXB*-R | CATTTGCGACACGGGCTAC |

**Table S5. Antibody list**

| **Western blot** | | |
| --- | --- | --- |
| N6-Methyladenosine | CST | 56593S |
| β-Actin | TransGen | HC201-02 |
| ZC3H13 | Abcam | ab70802 |
| Rabbit anti-CSF2 Polyclonal Antibody | absin | abs143539 |
| UNC5CL Polyclonal Antibody | Thermo | PA5-30917 |
| UNC5CL Polyclonal Antibody | Elabscience | E-AB-53207 |
| FoxJ1 Rabbit pAb | Immunoway | YT1751 |
| Rabbit Polyclonal Antibody to TNXB | IPODIX | ANP12378 |
| UPK3B polyclonal antibody | Bioworld | BS78298 |
| YTHDC1 Polyclonal antibody | Proteintech | 29441-1-AP |
| GAPDH (A531) polyclonal antibody | Bioworld | AP0066 |
| Lamin B1 polyclonal antibody | Bioworld | AP6001 |
| NF-kappaB p65 (D14E12) XP Rabbit mAb | Cell Signaling Technology | 8242 |
| Phospho-NF-kappaB p65 (Ser536) Rabbit mAb | Cell Signaling Technology | 3033 |
| IKB alpha Antibody | Abmart | T55026 |
| Phospho-I kappaB- alpha (Ser32/Ser36) Antibody | Abmart | TP56280 |
| **Immunohistochemical (IHC)** | | |
| ZC3H13 | Proteintech | 68526-1-1g |
| Anti-CD33 antibody | abcam | ab270942 |
| Anti-CD8 alpha antibody | abcam | ab209775 |
| [UNC5CL Antibody](https://www.novusbio.com/products/unc5cl-antibody_nbp2-24576) | Novus | NBP2-2457 |
| **Flow cytometry** | | |
| Fixable Viability Dye eFluor™ 780 | invitrogen | 65-0865-14 |
| CD16/CD32 Monoclonal Antibody (93) | Invitrogen | 14-0161-82 |
| CD45 Monoclonal Antibody (30-F11), eFluor 506 | invitrogen | 69-0451-82 |
| Brilliant Violet 510 anti-mouse CD3 Antibody | BioLegend | 100234 |
| CD4 Monoclonal Antibody (RM4-5), Super Bright 645 | Invitrogen | 64-0042-82 |
| CD25 Monoclonal Antibody (PC61.5), APC | Invitrogen | 17-0251-82 |
| FOXP3 Monoclonal Antibody (FJK-16s), PE | invitrogen | 12-5773-82 |
| CD8a Monoclonal Antibody (53-6.7), PerCP-eFluor 710 | Invitrogen | 46-0081-82 |
| Granzyme B Monoclonal Antibody (NGZB), APC | invitrogen | 17-8898-82 |
| IFN gamma Monoclonal Antibody (XMG1.2), PE | invitrogen | 12-7311-82 |
| Brilliant Violet 605™ anti-mouse/human CD11b | BioLegend | 101257 |
| CD11b Monoclonal Antibody (M1/70), Super Bright 780 | invitrogen | 78-0112-82 |
| F4/80 Monoclonal Antibody (BM8), eFluor™ 450 | Invitrogen | 48-4801-82 |
| CD206 (MMR) Monoclonal Antibody (MR6F3), APC | invitrogen | 12-2061-82 |
| CD86 (B7-2) Monoclonal Antibody (GL1), PE | Invitrogen | 12-0862-82 |
| CD11c Monoclonal Antibody (N418), APC | Invitrogen | 17-0114-81 |
| CD335 (NKp46) Monoclonal Antibody (29A1.4), PE | Invitrogen | 12-3351-82 |
| Atlantic Blue™ Anti-Mouse MHC Class II  (I-A/I-E) (M5/114.15.2) | Proteintech | AB-65122 |
| Ly-6G/Ly-6C Monoclonal Antibody (RB6-8C5), PerCP-eFluor 710 | Invitrogen | 46-5931-82 |
| Ly-6G/Ly-6C Monoclonal Antibody (RB6-8C5) | invitrogen | 17-5931-82 |
